# Serine/Threonine Protein Kinases as Attractive Targets for Anti-Cancer Drugs—An Innovative Approach to Ligand Tuning Using Combined Quantum Chemical Calculations, Molecular Docking, Molecular Dynamic Simulations, and Network-like Similarity Graphs

**DOI:** 10.3390/molecules29133199

**Published:** 2024-07-05

**Authors:** Magdalena Latosińska, Jolanta Natalia Latosińska

**Affiliations:** Faculty of Physics, Adam Mickiewicz University, Uniwersytetu Poznańskiego 2, 61-814 Poznań, Poland; magdalena.latosinska@amu.edu.pl

**Keywords:** kinase inhibitors, anti-cancer drugs, screening approach, molecular docking, novel approach, Structure-Binding Affinity Index, Structural-Binding Affinity Landscape

## Abstract

Serine/threonine protein kinases (CK2, PIM-1, RIO1) are constitutively active, highly conserved, pleiotropic, and multifunctional kinases, which control several signaling pathways and regulate many cellular functions, such as cell activity, survival, proliferation, and apoptosis. Over the past decades, they have gained increasing attention as potential therapeutic targets, ranging from various cancers and neurological, inflammation, and autoimmune disorders to viral diseases, including COVID-19. Despite the accumulation of a vast amount of experimental data, there is still no “recipe” that would facilitate the search for new effective kinase inhibitors. The aim of our study was to develop an effective screening method that would be useful for this purpose. A combination of Density Functional Theory calculations and molecular docking, supplemented with newly developed quantitative methods for the comparison of the binding modes, provided deep insight into the set of desirable properties responsible for their inhibition. The mathematical metrics helped assess the distance between the binding modes, while heatmaps revealed the locations in the ligand that should be modified according to binding site requirements. The Structure-Binding Affinity Index and Structural-Binding Affinity Landscape proposed in this paper helped to measure the extent to which binding affinity is gained or lost in response to a relatively small change in the ligand’s structure. The combination of the physico-chemical profile with the aforementioned factors enabled the identification of both “dead” and “promising” search directions. Tests carried out on experimental data have validated and demonstrated the high efficiency of the proposed innovative approach. Our method for quantifying differences between the ligands and their binding capabilities holds promise for guiding future research on new anti-cancer agents.

## 1. Introduction

### 1.1. Kinases—State of the Art

The human genome is estimated to contain between 19,000 and 20,000 protein-coding genes [1], of which 538 encode protein kinases [2,3] playing vital roles in controlling the behavior of each cell. The chemical activity of a protein kinase involves the removal of a phosphate group from high-energy adenosine triphosphate (ATP) and its covalent attachment to one of three amino acids with a free hydroxyl group. Phosphorylation results in a conformational transformation of the protein, which subsequently affects its binding capacity and activity (it activates/deactivates enzymes). Protein kinases are divided into two families, serine-threonine kinases (STKs, EC 2.7.11), and tyrosine kinases (TKs, EC 2.7.10) [4], depending on which protein residue is phosphorylated. STKs phosphorylate threonine or serine, while TKs phosphorylate tyrosine. The vast majority of protein kinases, at least 350, are serine/threonine kinases [5]. A number of kinases, so-called dual-specificity kinases, belong to both groups.

STKs are constitutively active, highly conserved, pleiotropic, and multifunctional kinases, which control several signaling pathways and regulate many cellular functions, such as cell activity, survival, proliferation, and apoptosis [6,7]. The dysregulation of their activity is a common cause of numerous diseases. Over the past decades, kinases have gained increasing attention as potential therapeutic targets, ranging from various hematologic and solid cancers (e.g., kidney, blood, brain, bladder, prostate, and ovaries) to viral diseases (HPV, malaria, and COVID-19). Protein kinase 2, formerly known as Casein kinase II (EC 2.7.11.1, CK2/CSNK2,), a ubiquitous and highly pleiotropic enzyme whose high constitutive activity plays an essential role in the development and intensification of the tumor phenotype, as well as in the transmission of infectious diseases, has been studied most intensively. Abnormally high levels of CK2 well documented in a number of leukemias (T-ALL, B-ALL, AML, CLL, CML), lymphomas (multiple myeloma and mantle cell), and cancers, including brain (glioblastoma and medulloblastoma), head and neck (squamous cell cancers), prostate, ovarian and cervical, mammary gland, lung, renal (cell carcinoma), bladder, pancreatic, cholangiocarcinoma, esophageal, gastric, hepatocellular, mesothelioma, melanoma, and other squamous cell carcinomas, support the observation that CK2 promotes cell survival by regulating oncogenes and plays an anti-apoptotic role [8,9,10]. It was discovered that the different mechanisms, including the prevention of caspase action [11] and potentiation of survival signaling, contribute to the global anti-apoptotic function of CK2 [12,13]. Therefore, CK2 inhibitors have been identified as a highly promising class of hematological and anticancer drugs. Recently, it has been discovered that CK2 can also be a target for neurological (autism, ADHD, schizophrenia, Poirier–Bienvenu neurodevelopmental syndrome, Alzheimer’s disease, Parkinson’s disease, and major depressive disorder), inflammation [14], and autoimmune (diabetic fibrosis, Crohn’s disease, and rheumatoid arthritis) [15] disorders. However, CK2 protein kinase seems to be one of the suitable therapeutic targets not only for the treatment of cancer or neurological disorders but also for viral infections, including β-CoVs [16,17,18]. CK2 was found to be upregulated dramatically in virus-infected cells. Several viruses use host cell CK2 to phosphorylate and modulate their own proteins. Once phosphorylated, these proteins support the viral life cycle through a variety of mechanisms. The CK2 signaling pathway has also been identified as being hijacked by SARS-CoV-2 [19]. It has recently been discovered that the SARS-CoV-2 nucleocapsid (N), playing an important role in the viral life cycle (replication, transcription, and genome packaging), binds to the stress granule proteins G3BP1/2 and to other mRNA-binding proteins of the host, including the protein kinase CK2 [20]. The inhibition of CK2 causes an endocytosis of the spike protein, which suggests that the antiviral effect involves the suppression of viral entry. Selective CK2 inhibitors are the hope for the treatment of many types of disease, in particular cancer and COVID-19. Therefore, CK2 remains and becomes an increasingly important target for broad-spectrum drug development.

A pivotal element in the identification and development of novel or repurposed pharmaceuticals is an understanding of the three-dimensional structure of the target in conjunction with the pathological process. The CK2 enzyme forms stable heterotetramers composed of two 44 kDa catalytic α-type subunits (α and/or α’) and two 26 kDa regulatory β-type subunits, which adopt a butterfly-shaped conformation. The acceptable forms of the so-called heterotetrameric holoenzyme are: ααββ, α′αββ, and α′α′ββ [21]. The α or α′ catalytic units, despite sharing a similar structural composition, are encoded by two different genes, *CSNK2A1* and *CSNK2A2*, respectively, while the CK2 regulatory subunit, β, is encoded exclusively by the *CSNK2B* gene. The α and α′ subunits phosphorylate the majority of the enzyme’s substrates, many of which are involved in gene expression and cell growth. The β subunit’s role is confined to regulatory functions such as the maintenance of enzyme cellular localization, stability, and substrate specificity. Both CK2α and CK2α′ subunits are involved in promoting tumor progression to a greater extent than CK2β. However, it is not possible to clearly determine what pro-oncogenic function each subunit plays, as it depends on the type of cancer, the presence of CK2 substrates and the isoform needed for their phosphorylation. Both the tetrameric and monomeric forms of CK2 are considered to be constitutively active and catalytically competent. The catalytic domain of CK2 consists of two parts, the β-rich N-terminal and α-helical C-terminal subdomains, and the active ATP site located in the cleft between them [22]. Due to the ATP site’s high degree of conservation, the selective inhibition of CK2 using active site inhibitors is an exceedingly challenging endeavor [22,23,24].

It has been established that certain small molecules have the capacity to inhibit the biological activity of CK2, thereby allowing the biological activity of CK2 to be controlled.

**Figure 1:**
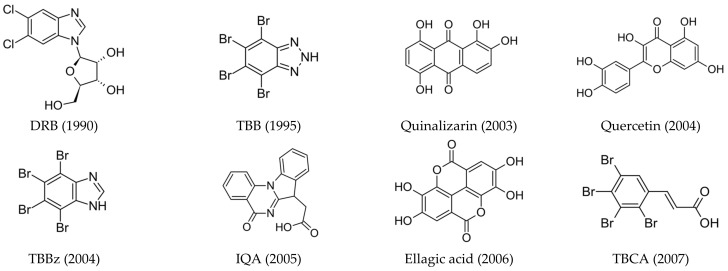

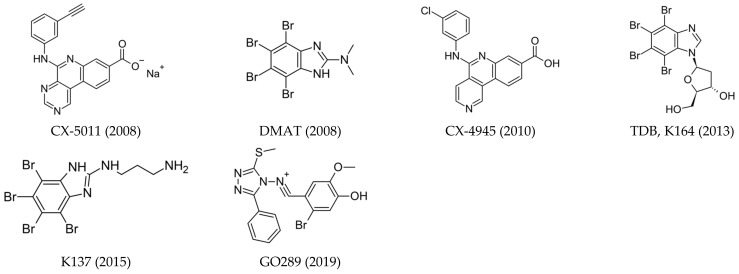
Structures of the most relevant CK2 inhibitors in chronological order of discovery. The date of discovery is given in brackets.

Although many kinase inhibitors have been discovered, only some of them, as seen in Figure 1, have shown significant therapeutic potential:

(1) **ATP-competitive inhibitors (targeting conserved orthosteric site):** coumarins (elagic acid [25]); carboxyl acid derivatives ([5-oxo-5,6-dihydroindolo-(1,2a)quinazolin-7-yl]acetic acid (IQA) [26,27], 2,3,4,5-tetrabromocinnamic acid (TBCA) [28], and CX-5011 [29]); polyhalogenated benzimidazoles (4,5,6,7-tetraiodo-1*H*-benzimidazole (TBI), 4,5,6,7-tetrabromo-1*H*-benzimidazole (TBBz), and 2-dimethylamino-4,5,6,7-tetrabromo-1*H*-benzimidazole (DMAT) [30]) and benzotriazoles (4,5,6,7-tetrabromobenzotriazole (TBB, TBBt) [31,32]); quinolones [33]; anthraquinones (Quinalizarin), xanthenones [34], and flavonoids (Quercetin) [35,36]; and hydroxycoumarines [37]), SRPIN803 derivatives [38,39], and bromoguaiacol derivatives of 1,2,4-triazole (GO289 [40]);
(2) **Allosteric inhibitors (targeting α/β interface, αD pocket, and interface between the glycine-rich loop and αC-helix):** 11-mer peptide (Pc) [41] and polyoxometalates (POMs) [42];
(3) **Bi-substrate inhibitors (each fragment targeting a different binding site)**: D-arginine-rich peptides (Arcs) [43] and N1-(4,5,6,7-tetrabromo-1*H*-benzimidazol-2-yl)-propane-1,3-diamine (K137) [44];
(4) **Dual and mutual binding ligands (targeting CK2/PIM, CK2/BRD4, CK2/SRPK1, CK2/HDAC1, CK2/Rio, and CK2/TNIK/DYRK1, respectively):** CPA, CPB, AMR, and SRPIN803, CX-4945 (Silmitasertib) [45], and 1-(β-D-2′-deoxyribofuranosyl)-4,5,6,7-tetrabromo-1*H*-benzimidazole (TDB, K164) [46,47];
(5) **Peptide-competitive inhibitors (substrate-competitive inhibitors):** CIGB-300 (CIGB-325) [48].

One of the most promising groups of compounds are the polyhalogenated benzimidazoles, which turned out to be a valuable scaffold that effectively competes with the ATP-binding site of CK2. The leading compounds 4,5,6,7-tetrabromo-benzotriazole (TBB) and 4,5,6,7-tetrabromo-1*H*-benzimidazole (TBI) show selectivity for one kinase, CK2, among more than 60 protein kinases tested, either Ser/Thr-specific or Tyr [31,49]. The aforementioned factor is of critical importance, given that kinases exhibit a high degree of sequence and structural similarity. (The lack of selectivity could potentially result in increased toxicity.) It was noted that, depending on the structure, polyhalogenated benzimidazoles downregulate native and recombinant CK2 from various sources, discriminate between different CK2 isoforms in vitro, inhibit other classical eukaryotic protein kinases (e.g., PIM, CDK9, DYRK, PKD, or atypical RIO1) with varying degrees of selectivity and potency, and induce apoptosis in numerous cancer cell lines (e.g., HeLa, CML, Mcl-1, and MCF-7). Although one of the most notable advantages of this class of compounds is their high selectivity, some of the polyhalogenated benzimidazole inhibitors are multi-targeted and may therefore become clinical candidates [49]. However, most of them, especially 4,5,6,7-tetrabromo- and 4,5,6,7-tetraiodo-1*H*-benzimidazoles, are polar compounds that do not penetrate cell membranes and therefore, although they exhibit strong in vitro activity as CK2, PIM-1, DYRK1A, or RIO1 inhibitors, they do not have medical applications. It was found that glycone (2′-deoxyribose) addition improves their solubility and ensures effective intercellular transport [47].

On the other hand, one of the oldest known CK2 kinase inhibitors, 5,6-Dichloro-1-β-D-ribofuranosylbenzimidazole (DRB), has been identified as an inhibitor of CDK9 [50], playing a crucial role in the regulation of transcription. Its dysregulation has been implicated in various diseases, including cancer, cardiovascular disorders, and viral infections. DRB induces apoptosis in breast cancer cells by inhibiting the expression of Mcl-1 [51]. Its brominated analogue 1-(β-D-2′-deoxyribofuranosyl)-4,5,6,7-tetrabromo-1*H*-benzimidazole (K164) [46,47], whose cytotoxic effectiveness involves inducing apoptosis through the parallel inhibition of CK2 and PIM-1, is highly promising. Moreover, it affects CK2 activity more persistently than CX-4945 [45], which is crucial for cellular processes such as cell survival, proliferation, and migration. K164 induces apoptosis in cervical cancer (HeLa), chronic myeloid leukemia (CML) [47,52], and some types of breast cancer [37]. The most potent kinase inhibitor among benzimidazoles, 4,5,6,7-tetraiodobenzimidazole (TIBI), additionally inhibits atypical kinase RIO1 [53], which is essential for cell viability, growth, and division, as well as genomic stability.

### 1.2. Research Motivation and Concept

Nevertheless, despite the accumulation of a vast amount of experimental data, there is still no “recipe” that would facilitate the search for new effective kinase ligands. So far, only one factor has been observed that may prove helpful in the search for more effective inhibitors: the presence of the halogen atoms on the benzene ring of these heterocycles. This has proven to be a key factor in filling the CK2 hydrophobic pocket at the ATP-binding site [33,54] and stabilizing complexes of CK2 with their inhibitors [55,56]. The extent to which the protein pocket is occupied is undoubtedly an important factor, although it may not be sufficient. The two fundamental requisites for inhibitors are high affinity and high selectivity. High affinity is determined by the involvement of specific interactions and functional groups in the binding efficiency. High selectivity is difficult to achieve, especially in the case of such large families of structurally and functionally related proteins as kinases, and requires chemical modifications that increase affinity for the target and decrease affinity for the non-target and/or decrease affinity only for unwanted targets.

In the light of the above, the aim of our study was to propose an effective screening method that would be useful for checking how ligand modification affects its ability to form intermolecular bonds and, consequently, its binding affinity and biological activity.

The foundation for the development of our novel approach was the Similar Property Principle (SPP) [57], which states that structurally similar molecules tend to have similar properties. It seems that incremental alterations to the chemical structure of a ligand which is an efficient inhibitor should be sufficient to maintain its biological activity against a specific target.

**Figure 2:**
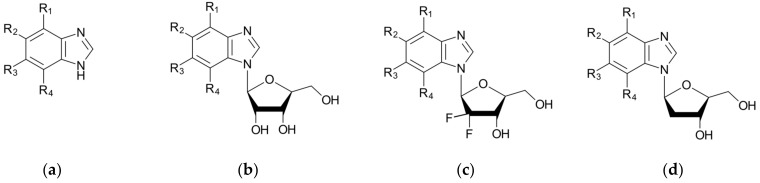
The ligand structures of interest that are being screened against a target protein CK2. The ligands of each series (**a**–**d**) differ in both aglycone, for which the substituents (R_1_, R_2_, R_3_, R_4_—halogen/hydrogen atoms) are indistinguishably altered, and the glycone moiety, which is modified at the 2′ position.

Therefore, we chose three series of ligands differing in the glycone moiety (ribose, 2′-deoxyribose, and 2′-deoxyribose,2′2′-difluoro-ribose), in which the substituents (R_1_, R_2_, R_3_, and R_4_—halogen/hydrogen atoms) are indistinguishably altered, see Figure 2. Sugar modification in 2′-deoxyribose-2′2′-difluoro-ribose has been shown to have significant efficacy in the treatment of cancer. It has led to a substantial improvement in the efficacy of the original Cytarabine, and to the development of Gemcitabine. A recent study has analyzed the differences between the Cytarabine and Gemcitabine binding modes [58].

The widely used technique in drug design that allows for the prediction of the binding of the ligand in the active site of a specific therapeutic target and thus, the screening of ligands, is molecular docking [58,59,60]. However, the analysis of docking results is typically limited to the different docking scores or binding affinity (BA), which describe the strength of the protein–ligand interaction. Unfortunately, this scalar parameter does not provide detailed insight into the nature of the binding. The factors that determine the strength of protein–ligand binding are based on specific interactions, and their isolation and understanding is of key importance in drug screening [58].

Therefore, in this paper, two new approaches are proposed:

(1) The heatmaps approach, which has proven to be highly effective in
  - Identifying the strongest binding components as well as obstacles in the ligands;
  - The visualization of the flexibility/dynamics of protein residues.
(2) The Structure Binding Affinity Landscape approach, based on the novel Structure-Binding Affinity Landscape Index (SBAI), that helped measure the degree to which binding affinity gained from molecular docking is gained or lost in response to a relatively small change in the ligand structure.

Tests conducted on the set of polyghalogeno-1*H*-benzimidazoles (already known and newly designed) confirmed and demonstrated the high effectiveness of the proposed unique approach. The comparison of the selected ligands, presented in this paper, demonstrates the efficacy of the novel methodology. The combination of physicochemical and structural properties responsible for the effective inhibition of kinases helped discover “dead” and “promising” research directions.

The protocol proposed in our paper show promise for virtually screening ligands and effectively searching for drug improvements.

## 2. Results

### 2.1. Physicochemical Profile (ADME) and Key Pharmacokinetic Parameters of the Ligands

The physicochemical profile parameters that describe the physicochemical profile and pharmacokinetics behavior as well as the toxicity of the CK inhibitors estimated using different ADME (Absorption, Distribution, Metabolism, and Excretion) and PAMPA (Parallel Artificial Membrane Permeability Assay) [61,62,63] protocols are listed in Supplementary Tables S1–S3, respectively.

The molecular weights of the ligands range from 321 to 773 g/mol and some of them exceed 500 g/mol. The predicted lipophilicity, consensus LogP, for the studied ligands ranges from 0.10 to 3.66. Therefore, they all show high and positive lipophilicity in the range of 1–4, optimal for drugs. It appears that the increase in lipophilicity caused by the substitution of two fluorine atoms to 2′-deoxyribose should have the potential to modify hydrophobic targets. The substitution of two fluorines at the 2′ position of the 2′-deoxyribose due to the strong electron-withdrawing nature of fluorine, its weak polarizability, and its ability to expand the hydrophobic domain should be advantageous. The ligands show no Pan Assay of Interference Structures (PAINSs). Nevertheless, the presence of halogen atoms gives rise to the emergence of structural alerts (BRENK).

QED (a measure of drug-likeness based on eight drug-likeness related properties, including molecular weight (MW), lipophilicity (log P), the number of hydrogen bond acceptors (N_HBA_), the number of hydrogen bond donors NH_BD_, polar surface area (PSA), stereo-specificity (N_rotb_), the number of aromatic rings (NAr), and the number of alerts for undesirable functional groups (ALERTs)) is lower for polyhalogenated-1*H*-benzimidazoles substituted with a glycone moiety than parental ones. It exceeds 0.67 only for 5,6-dibromo- and 5,6-dichloro- derivatives, because the iodine-containing derivatives do not satisfy the golden triangle rule (200 ≤ MW ≤ 50; −2 ≤ logD ≤ 5). Nevertheless, none of the ligands under investigation violate the lead-likeness rules, and PAINSs, one of the most significant filters, does not exclude any of them. The predicted water solubility index suggests that all of the candidate ligands should be highly water soluble. The predicted synthetic accessibility score (SAS) values for the ligands do not exceed 5.10, indicating that these compounds can be synthesized relatively easily. Abbot’s bioavailability score for all ligands placed them within the 55% probability class. The topological polar surface area (TPSA), which describes the passive transport of molecules through membranes, is 20 units higher for ligands with ribose at the N(1) position than for the others, but still does not exceed 140 units, so it is at the optimal level.

Most of the ligands are expected to cross the blood–brain barrier (BBB). The PAMPA describing the permeability of substances from a donor compartment through a lipid-infused artificial membrane into an acceptor compartment, strongly depends on pH. CK2 kinase shows the best enzyme activity at pH 7.4. The modification of the glycone moiety at the 2′ position, removal of –OH, or substitution of two F atoms, causes a significant increase in the PAMPA at pH = 7.4, but results in a negligible modification at pH = 5.0. The substitution of the glycone moiety with two fluorine atoms at 2′ position of the 2′-deoxyribose ring results in the formation of ligands with moderate to high levels of PAMBABBB. The gastrointestinal absorption (GI), a key parameter in assessing the in vivo performance of an orally administrated drug formulation, is high for all studied ligands. Moreover, most of the ligands can be substrates of the multidrug resistance protein, permeability glycoprotein (PGP). The ability of PGP to alter the pharmacokinetic profiles of substrates is considered a key factor in worsening treatment outcomes. This result may be attributed to the lipophilic properties of the ligands. The substitution of multiple Br atoms increases plasma protein binding (PPB), which is known to result in a low therapeutic index. Furthermore, the presence of Br atoms has been associated with an increased risk of drug-induced liver injury (DILI). However, the risk scores for hepatotoxicity (H-HT) and the probability of genetic toxicity (AMES assay) in humans are relatively low. Selected ligands, including 4,5,6,7-tetraiodo-, 5,6-diiodo-, and 5,6-diiodo,4,7-dibromo-, have the highly desired very low carcinogenicity.

It should be emphasized that there is no set of ideal pharmacokinetic parameters that a given inhibitor should exhibit, as it depends on the specific target requirements. The ligands that appear to have an optimal pharmacological profile (achieve the desired biological activity with minimal side effects) can be selected based on ADME analysis. Nevertheless, candidate ligands are similar in their physicochemical properties to the already known inhibitors.

### 2.2. Target Binding Site and 3D Pharmacophore Analysis

The crystal structures of three targets, CK2α (PDB code: 4KWP [35] and 8AEC [64]), PIM-1 (PDB codes: 4DTK [49] and 5KGD [65]), and RIO1 (PDB code: 3RE4) [66] with the highest available resolutions (1.25 and 1.09 Å, 1.86 and 1.98 Å, and 2.0 Å, respectively) were retrieved from the Protein Data Bank, PDB (http://www.rcsb.org/pdb/, accessed on 29 March 2024).

Protein alignment provides a global comparison of the percentage of identity/similarity/gaps across the entire sequence. The CK2α and PIM-1 protein sequences differ by 24.59/43.44/35.52%, while the CK2α and RIO1 sequences differ by 23.34/36.87/45.59%. The CK2α and PIM-1 protein sequences differ by 24.59/43.44/35.52%, while the CK2α and RIO1 sequences differ by 23.34/36.87/45.59%. PIM1/RIO similarity is even lower, at 19.26/37.71/51.27%. PIM-1 and RIO1 similarity is low, at 19.26/37.71/51.27%.

A pocket search and 3D pharmacophore analysis were carried out using CavityPlus [67], Table 1.

All structures exhibit optimal pK_d_ ligandability properties in their main cavities. However, the predicted average pK_d_ in 8AEC is lower than six. The DrugScore for major cavities in CK2α (4KWP and 8AEC) and PIM-1 (4DTK and 5KGD) is greater than 600. Consequently, the CK2α and PIM-1 kinases are highly susceptible to therapeutic modulation by drugs. In contrast, the druggability of RIO1 (3RE4) is only moderate, indicating that the RIO1 target can be weakly modulated by drugs. Interestingly, the pharmacophores in 4KWP, 8AEC and 5KGD are hydrophobic, while 4DTK and 5KGD are electrostatic.

**Figure 3:**
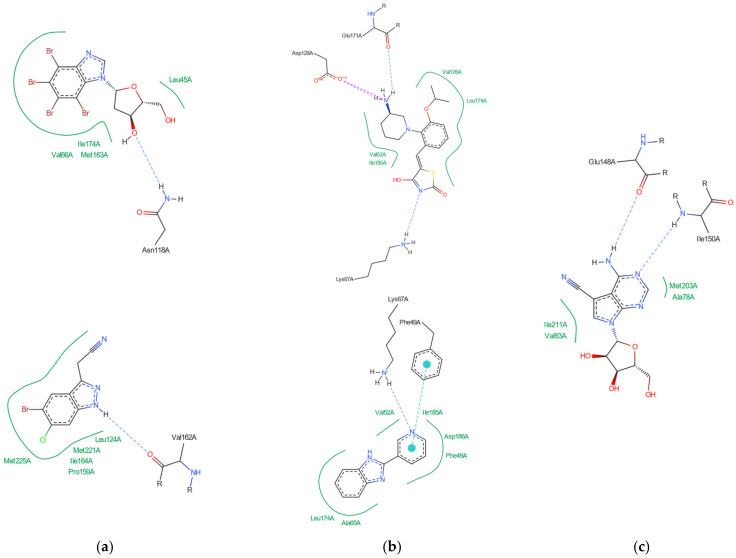
Key protein–ligand interactions in the (**a**) CK2α (4KWP—top and 8AEC—bottom), (**b**) PIM-1 (4TDK—top and 5KGD—bottom), and (**c**) RIO1 (3RE4) structures (hydrophobic contacts in green, ionic interactions in pink, hydrogen bonds in blue, and stacking interactions in cyan).

In-depth analysis of the 4KWP, 8AEC, 4DTK, 5KGD, and 3RE4 co-crystals, Figure 3, reveals the key interactions between the native ligand and target.

In the 4KWP complex one nearly linear NH⋯O hydrogen bond of 3.14 Å linking O from the 2′-position in the ligand (4,5,6,7-tetrabromo-1-(2-deoxy-beta-D-erythro-pentofuranosyl)-1*H*-benzimidazole) and N from Asn118, one long and highly non-linear OH⋯O bond of 3.81 Å and 102.05° linking the ligand with Asn118, and one strong and nearly linear Br⋯O halogen bond of 3.0 Å and 172.68° linking Br at the 7-position of the ligand’s ring with Val116 were found. Additionally, two long water bridges with Arg43 and Leu45, of 4.06 and 3.91 Å stiffen the structure. A few hydrophobic contacts link the ligand with protein residues: Ile174, Val66, Met163, and Leu45. In 8AEC, one nearly linear N–H⋯Cl bond of 3.95 Å linking Cl from ligand (2-(5-bromanyl-6-chloranyl-1*H*-indazol-3-yl)ethanenitrile) and N from Met137 was found. Furthermore, a few hydrophobic contacts link the ligand with residues: Val 162, Ile 164, Leu124, Met221, Met222, Leu124, Tyr125, and Leu128. 5-bromanyl-6-chloranyl-1*H*-indazol-3-yl)ethanenitrile is an allosteric inhibitor.

In 4DTK, four nonlinear hydrogen bonds link Lys67 (N–H⋯N of 2.91 Å), Asp128, Glu171, and Asp186 (N–H⋯O of 2.93, 3.30 and 2.92 Å) to ligand ((5Z)-5-{2-[(3R)-3-aminopiperidin-1-yl]-3-(propan-2-yloxy)benzylidene}-1,3-thiazolidine-2,4-dione). Additionally, Leu44, Phe49, Val52, Ala65, Leu174, and Ile185 form hydrophobic interactions of 3.86, 3.95, 3.92, 3.47, 3.93/3.58, and 3.90 Å, respectively. In 5KGD, only one nonlinear hydrogen bond links Lys67 (N–H⋯N of 2.91 Å), to the native ligand (2-pyridin-3-yl-1~{*H*}-benzimidazole). Additionally, Asp186, Phe49, Leu174, Ala65, and Leu44, form hydrophobic interactions of 3.50, 3.55, 3.51, and 3.85 Å, respectively. The selection of 5KGD for further analysis was based on its pharmacophore similarity to 4KWP.

In 3RE4, hydrogen bonds N–H⋯O Glu148 of 3.17 Å and 166.77°, N–HN of 3.07 Å and 169.57° with Ile150, and two very long and weak O–H⋯O and O–H⋯N hydrogen bonds link the native ligand (4-amino-7-(beta-D-ribofuranosyl)-7*H*-pyrrolo[2,3-d]pyrimidine-5-carbonitrile) with Asp212. Furthermore, the ligand participates in the hydrophobic interactions with Met203, Ala78, Ile211, and Val63.

Each binding pattern is different, which is not surprising given the low similarity of the studied targets (about 24%) and different ligands, but a certain regularity can be observed: in each case, hydrophobic interactions dominate, but in the case of PIM-1 and RIO1, the strong hydrogen bonds are of significant importance.

### 2.3. Molecular Docking Results

All target proteins were prepared according to standard protocols. The protocol used to dock ligands, shown in Figure 2a–d, to the particular proteins (CK2α, PIM-1, or RIO1) was closely related to that previously described [58,59]. The water molecules and native ligand that co-crystallized with protein were removed and the protonation state of the protein was corrected before docking. A pocket/cavity was identified in each target. (The size of the pocket, its geometry, and its hydrophobicity, which are crucial in predicting protein–ligand bonding, were quantified, as shown in Table 1).

The pocket containing native ligand in 4KWP has a surface area of 1864.31 Å^2^, a volume of 1438.21 Å^3^, and thus a surface/volume ratio of 1.30. Its hydrophobicity is 0.59. The pocket containing native ligand in 8AEC is very small. This allosteric cavity has a surface area of 346.64 Å^2^, a volume of 297.98 Å^3^, and thus a surface/volume ratio of 1.16. Its hydrophobicity is much lower and does not exceed 0.76. Most studied ligands seem too large for this cavity, the only exceptions are two derivatives 5,6-dibromo- and 5,6-dichloro-.

The pocket containing the native ligand in 4DTK is much smaller and has a surface area of 701.04 Å^2^, a volume of 673.28 Å^3^, and thus a surface/volume ratio of 1.04. Its hydrophobicity is a bit higher at 0.66. The pocket containing native ligand in 5KGD has a surface area of 693.53 Å^2^, a volume of 429.23 Å^3^, and thus a surface/volume ratio of 1.45. Its hydrophobicity is also 0.66.

The pocket containing native ligand in 3RE4 is slightly larger than those in 4DTK. It has a surface area of 721.66 Å^2^, a volume of 698.37 Å^3^, and thus a surface/volume ratio of 1.03. Its hydrophobicity is also 0.66.

The active site and the search space was defined as a subset of approximately 9.0–15.0 Å. To validate the docking protocol, it is necessary to determine the feasibility of reproducing the experimental binding position. This allows the most appropriate docking procedure to be selected and new ligands to be successfully docked. Test re-docking of the native ligand was performed using the same docking protocol that would then be used for the actual docking. The pose’s root-mean-square deviation (RMSD) from its conformation in the parent structure was 0.112 Å and easily met the RMSD < 3 Å criterion. Consequently, the same docking protocol was used for all ligands.

#### 2.3.1. Protein Kinase CK2α Target

**Figure 4:**
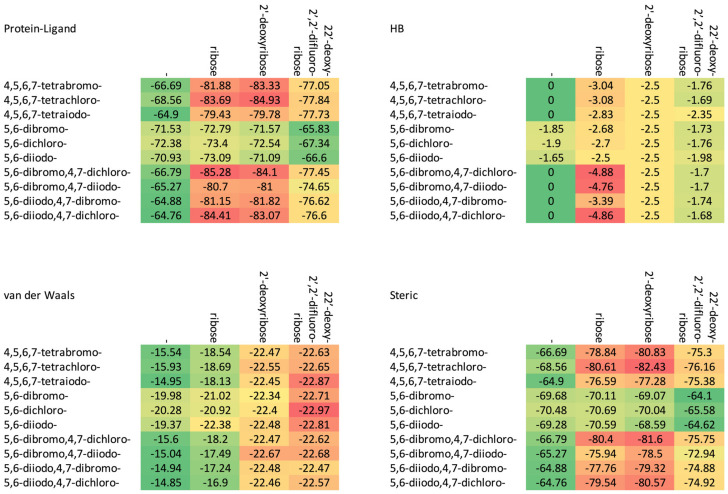
A comparison of the van der Waals, steric, hydrogen bond, and protein–ligand binding energy in four series of ligands. (The numerical data in kcal/mol are listed in Table 2.) The red–yellow–green scheme, with dark red indicating strong interactions and dark green indicating very weak ones, was applied.

**Figure 5:**
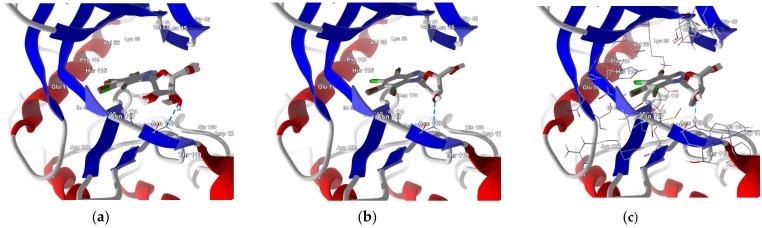
The best poses for three series of the ligands differing by glycone moiety: (**a**) ribose, (**b**) 2′-deoxyribose, and (**c**) 2′-deoxy-2′,2′-difluoro-ribose. The hydrogen bond between the ligand and the Asn118 residue of CK2α is shown with a blue dashed line.

The ligands were prepared and docked to the binding site in CK2α, which had been previously prepared by correcting protonation and atomic hybridization. The docking results are summarized in Table 2 and Figure 4. The best poses that led to the stabilization of the complex with the highest binding/docking score are shown in Figure 5.

The increase in the binding affinity of the ligands with the 2′-deoxy-2′,2′-difluoro-ribose moiety substituted at N(1) of the polyhalogeno-1*H*-benzimidazole ring is clearly evident. The protein–ligand binding energy is found to be strongly dependent on contributions from steric interactions and hydrogen bonds, while the binding affinity is found to be strongly affected by the van der Waals interactions. The heatmaps shown in Figure 4 help to reveal these relationships. Both the halogens of the aglycone and glycone moieties play a crucial role in the ligands under study as they significantly influence the electron density distribution within the ligand.

#### In-Depth Analysis of the Native Ligand Binding Mode

It is well known that ATP binding is typically mediated by residues that are conserved across species (lysine, aspartate, and glycine) and interact with the phosphate groups of ATP. Previous structural studies have demonstrated that the highly acidophilic protein kinase CK2α binding site is composed of Val66, Phe113, Val 51, Leu59, and Val116 residues [67]. However, when bound to polyhalogeno-1*H*-benzimidazoles, the nonpolar hydrophobic residues Val66 and Ile174 were found to play a key role in the catalytic pocket of CK2α. Moreover, mutating these residues to smaller ones, e.g., alanine, makes CK2α much less sensitive [68,69].

An in-depth analysis of the protein–ligand binding mode performed from the perspective of the residues and the ligand itself can help in understanding the nature of the binding mode. The first approach permits the comprehensive analysis of the total binding mode, whereas the second approach permits the role of individual ligand substituents to be investigated and the importance of the individual atomic position to be assessed.

#### Residues Perspective

**Figure 6:**
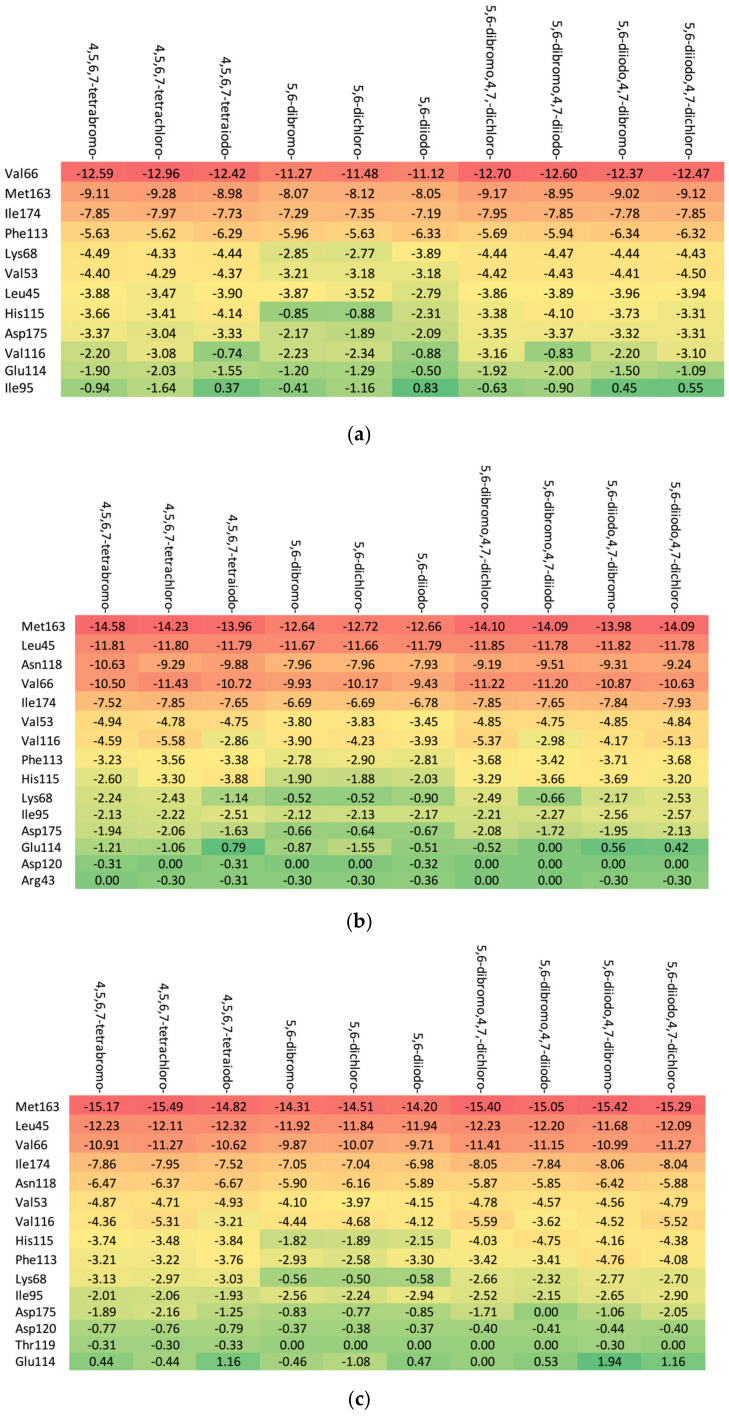

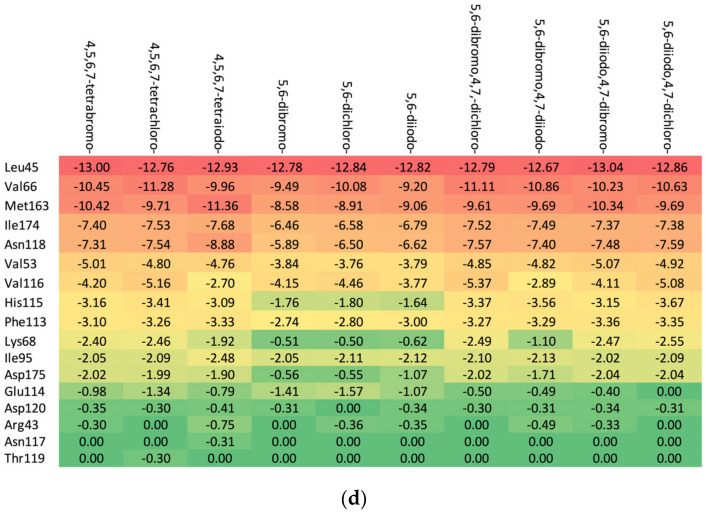
The binding strength of the ligands (**a**) without glycone and with different glycone moieties, (**b**) ribose, (**c**) 2′-deoxyribose, and (**d**) 2′-deoxy-2′,2′-difluoro-ribose to individual residues. The heatmaps visualize the binding mode in a red–yellow–green scheme, with dark red indicating strong interactions and dark green indicating very weak ones (kcal/mol units).

The in-depth inspection of the protein–ligand binding mode revealed that it remains consistent among all studied ligands that bind to 15–17 CK2α residues depending on the particular aglycone moiety (ribose: Met163, Leu45, Asn118, Val66, Ile174, Val53, Val116, Phe113, His115, Lys68, Ile95, Asp175, Glu 114, Asp120, and Arg43; 2′-deoxyribose: Met163, Leu45, Val66, Ile174, Asn118, Val53, Val116, His115, Phe113, Lys68, Ile95, Asp175, Asp120, Thr119, and Glu114; and 2′-deoxy-2′,2′-difluoro-ribose: Leu45, Val66, Met163, Ile174, Asn118, Val53, Val116, His 115, Phe113, Lys68, Ile95, Asp175, Glu114, Asp120, Arg43, Asn117, and Thr119), as shown in Figure 6. The residues that distinguish the series are weakly ligand-bound. Ligand analogues, not substituted with a glycone moiety (i.e., polyhalogeno-1*H*-benzimidazoles), bind up to 12 residues (4,5,6,7-tetrabromo-1*H*-benzimidazole: Val66, Ile174, Met163, Phe113, Lys68, Val53, Asp175, Val116, Leu45, and His115; and 4,5,6,7-tetraiodo-1*H*-benzimidazole: Val66, Met163, Ile174, Phe113, Lys68, Val53, His115, Leu45, and Asp175), as shown in Figure 6. Unlike ligands containing a glycone moiety, parental polyhalogenated-1*H*-benzimidazoles interact with the Phe113 residue.

The heatmaps visualization, shown in Figure 6, permits the assessment of how alterations in the binding specifically change the binding efficacy of individual protein residues with the ligand.

The residues exhibiting the strongest binding affinity are presented in descending order, as follows:

(a) Ribose-: Met163 > Leu45 > Asn118 > Val66 > Ile174 > Val53.
(b) 2′-deoxyribose-: Met163 > Leu45 > Val66 > Ile174 > Asn118 > Val53.
(c) 2′-deoxy-2′,2′-difluoro-ribose-: Leu45 > Val66 > Met163 > Ile174 > Asn118 > Val53.

Although each ligand interacts with many residues of CK2α, only Met163, Leu45, Asn118, Val66, Ile174, and Val53 are of major importance, as shown Figure 6. The strongest binding between the ligand and Leu45 occurs when the substituent at the N(2) position of the polyhalogeno-1*H*-benzimidazole ring is ribose, while the strongest binding between the ligand and Met163 occurs when the substituent is 2′-deoxy-2′,2′-difluoro-ribose. In both cases, the presence of identical halogens in all four positions (R_1_, R_2_, R_3_, and R_4_) leads to an increase in bond strength.

The binding mode of each ligand can be treated as a specific “binding fingerprint”. Various mathematical indices reveal differences in the binding patterns of specific ligands compared to the actual 1-(β-D-2′-deoxyribofuranosyl)-4,5,6,7-tetrabromo-1*H*-benzimidazole (K164) ligand. Manhattan distance measures the absolute difference between variables, Euclidean distance uses the squared difference, and the simple additive method shows the balance of contributions, Table S4.

The 4,6-dichloro-, 5,6-dibromo-, and 5,6-diiodo- derivatives show the most significant differences in their binding modes compared to the reference ligand, especially in the binding with the two main hydrophobic residues Val66 and Lys174. The docking results suggest that among the candidate ligands, the 4,5,6,7-tetraiodo- and 5,6-dibromo,4,7-dichloro- from the 2′-deoxy-2′,2′-difluoro-ribose series or 5,6-dibromo,4,7-dichloro- from the ribose series may be a very good alternative to the native 5,6,7,8-tetrabromo- ligand.

The 2′-deoxy-2′,2′-difluoro- derivatives have the highest binding affinity, shown in Table 2 and Figure 4 and Figure 6, among the studied ligands. Thus, a 2′,2′-difluoro modification of glycone seems highly promising and worth further research.

#### Ligand Perspective

The manner in which the ligand binds to its target, analyzed from its own perspective, allows us to ascertain the role of individual atoms in relation to the target. Manhattan, Euclidean, and additive distances between the native and studied ligand are listed in Table S5. Halogen atoms play a pivotal role in the filling of the target pocket; therefore, their contribution was analyzed separately.

Although all atoms of the ligand interact with CK2α, only some of them play a key role in this process. The most significant differences in binding strength compared to the reference ligand occur at the 4,5,6,7 positions of the 1*H*-benzimidazole ring and the unsubstituted nitrogen N and C(2) in the benzimidazole ring, as well as oxygen from the –CH_2_OH group of glycone. From the ligand perspective, the most promising are 4,5,6,7-tetraidio- and 5,6-dibromo,4,7-dichloro- from the 2′-deoxy-2′,2′-difluoro- ribose series.

#### 2.3.2. Proto-Oncogene Serine/Threonine-Protein Kinase PIM-1 Target

Dual-ligand targeting drugs are considered as a promising tool to increase the specificity of chemotherapy. Therefore, an investigation was conducted to ascertain whether the most promising ligands have the potential to become dual ligands. The most promising ligands, 2′-deoxy-2′,2′-difluoro-ribose derivatives, were docked to the binding site in PIM-1 kinase (5KGD), which had been previously prepared by protonation and atomic hybridization correction. The docking results are summarized in Table 3.

**Figure 7:**
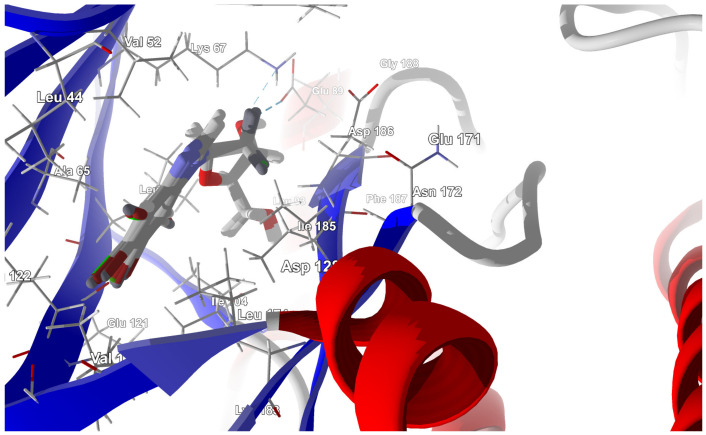
The best poses for the series of the 2′-deoxy-2′,2′-difluoro-ribose ligands. The hydrogen bonds between the ligand and the Lys67 and Glu89 residue of PIM-1 are shown with a blue dashed line.

The best poses that led to the stabilization of the complex with the highest binding/docking score are shown in Figure 7.

**Figure 8:**
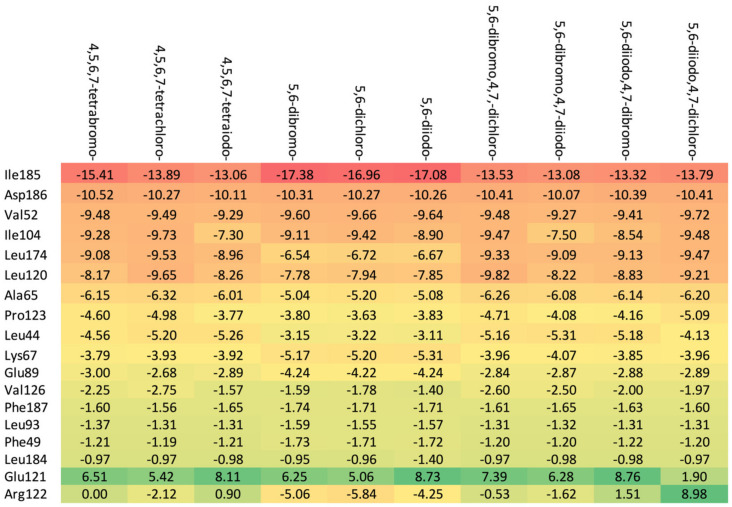
A comparison of the key protein–ligand interactions using a heatmap (target 5KGD), which visualizes the binding mode in a red–yellow–green scheme, with dark red indicating strong interactions and dark green indicating very weak ones (kcal/mol units).

As shown in Table 3, the higher the halogen content, the higher the binding affinity. For 4,5,6,7-tetrabromo- and 5,6-diiodo,4,7-dichloro-, the highest affinity was observed. The heatmap, Figure 8, is used in the assessment of the alterations in the binding strength resulting from changes in both halogen number and type.

The ordering of the strongest binding residues in descending order of binding strength is as follows: Ile185 > Asp186 > Val52 > Ile104 > Leu174 > Ala65 > Pro123. The heatmap shown in Figure 8 is also highly effective in identifying these obstacles in the ligands. Although the overall binding mode, including binding affinity, appears to be satisfactory, the ligands are strongly repelled from Glu121 or Arg122 (5,6-diiodo- and 4,7-dichloro-) by the halogen atom at the R_3_ position.

#### 2.3.3. Atypical Protein Kinase RIO1 Target

Protein kinase CK2 was identified as a partner of RIO1 (CK2-mediated phosphorylation regulates RIO1 activity) [53]. Therefore, the potential use of the ligands under investigation as the dual inhibitors of both CK2 and atypical RIO1 has also been explored.

The ligands with a 2′-deoxy-2′,2′-difluoro-ribose moiety were docked to the binding site in RIO1 (3RE4). A protonation and hybridization correction were previously used in the preparation of the binding site and ligands. The docking results are summarized in Table 4.

**Figure 9:**
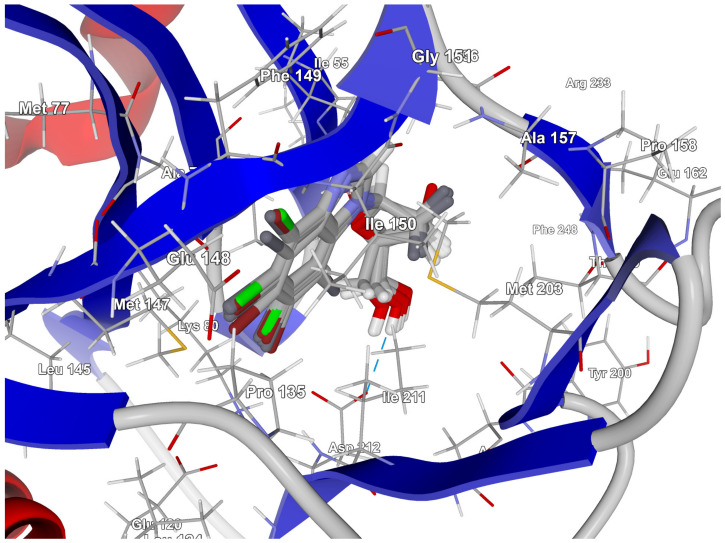
The best poses for the series of the 2′-deoxy-2′,2′-difluoro-ribose ligands. The hydrogen bond between the ligand and the Ile211 residue is shown with a blue dashed line.

The best poses that led to the stabilization of the complex with the highest binding/docking score are shown in Figure 9.

The highest binding affinity for 4,5,6,7-tetrabromo-, 5,6-dibromo,4,7-dichloro-, and 5,6-diiodo,4,7-dichloro- is clearly visible. The smaller contributions from van der Waals interactions, and larger contributions from hydrogen bonds, observed in 5,6-dihalogeno- derivatives result in high protein–ligand binding energies, yet the binding affinities are weak.

**Figure 10:**
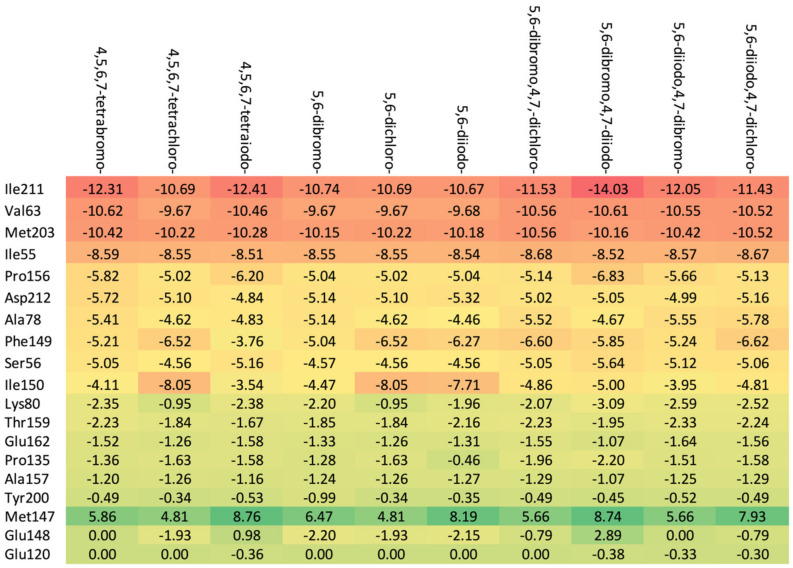
A comparison of the key protein–ligand interactions using a heatmap (target 3RE4), which visualizes the binding mode in a red–yellow–green scheme, with dark red indicating strong interactions and dark green indicating very weak ones (kcal/mol units).

A comparison of the heat maps indicates that although the overall binding affinity appears to be satisfactory, the ligand is strongly repelled from Met147 by the halogens at the R_1,_ R_2,_ and R_4_ positions, as shown in Figure 10. This effect is not apparent for a single binding affinity parameter. As can be observed, the heatmaps are highly effective in identifying the strongest components of the binding (indicated in dark red) as well as the obstacles in ligands.

### 2.4. Protein Flexibility and Molecular Dynamics Simulations

One of the disadvantages of the molecular docking approach is the assumption of a rigid or quasi-rigid ligand. Even the assumption of the flexibility of the ligand/residue in the active site does not necessarily reflect the mobility of the entire protein. The combined B-factor analysis and Molecular Dynamics Simulations, MDSs, have the potential to considerably enhance MD analysis in this area, thus providing a means of validating its accuracy.

The fundamental question is whether active sites exhibit flexibility. In most cases, it is only the backbone residues that are flexible, while the active site residues are rigid.

**Figure 11:**
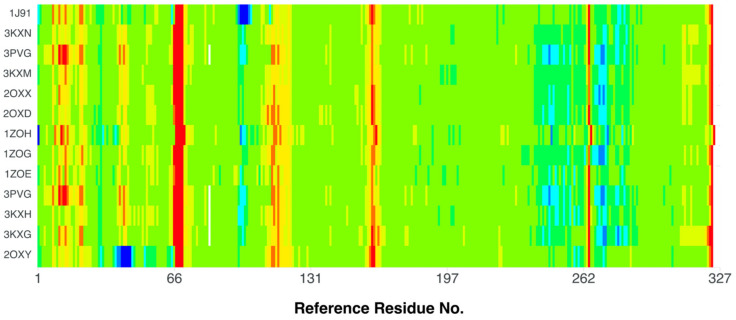
A comparison of the differences between the normalized B-factors (Debye–Waller factor or atomic displacement parameters) of different CK2 structures containing 4,5,6,7-tetrabromo-, (3KXM, 2OXY, 2OXX, 2OXD, 1ZOH, 1ZOG, 1ZOE, 3PVG, 3KXH, and 3KXG), 4,5,6,7-tetraiodo-1*H*-benzimidazole (3KXN), and 4,5,6,7-tetrabromobenzotriazole (1J91) ligands (organism: *Zea mays*, expression system: *Escherichia coli*) in relation to the best-quality structure, containing 1-(β-D-2′-deoxyribofuranosyl)-4,5,6,7-tetrabromo-1*H*-benzimidazole (K164) as the ligand (4KWP; organism: *Homo sapiens*). The more rigid fragments are indicated in red, while the less rigid ones are indicated in blue.

The comparison of the stiffness of different CK2 structures containing 4,5,6,7-tetrabromo-, (3KXM, 2OXY, 2OXX, 2OXD, 1ZOH, 1ZOG, 1ZOE, 3PVG, 3KXH, and 3KXG [49,54,70]), 4,5,6,7-tetraiodo-1*H*-benzimidazole (3KXN [49]), and 4,5,6,7-tetrabromobenzotriazole (1J91 [71]) ligands (organism: *Zea mays*, expression system: *Escherichia coli*) in relation to the best-quality structure, namely 4KWP (organism: *Homo sapiens*), Figure 11, indicates an increased rigidity of residues 65–69 and 160–161 in all structures. On the other hand, the rigidity of the residues 39–45, and 97–102 has decreased in 2OXY and 1J91, respectively.

The 2OXY (ligand: 4,5,6,7-tetrabromo-1*H*-benzimidazole) is more flexible than 3KXN (ligand: 4,5,6,7-tetraiodo-1*H*-benzimidazole) in the region of three residues Lys49, Asn61, and Val73; the 2OXX (ligand: 4,5,6,7-tetrabromo-1*H*,3*H*-benzimidazol-2-thione) is more rigid than 2OXY in the region of the Lys49, Asn61 and Pro72 residues; and 1ZOG (ligand: 4,5,6,7-tetrabromo-2-(methylsulfanyl)-1*H*-benzimidazole) is more rigid than 2OXY in the Lys49, Asn61 and Val73 regions. Figure 11 illustrates the differences in flexibility between the 2OXY and 4KWP structures. In the catalytic loop, the stiffness increases significantly in 4KWP due to the presence of additional bonds between the glycone and the target residues.

**Figure 12:**
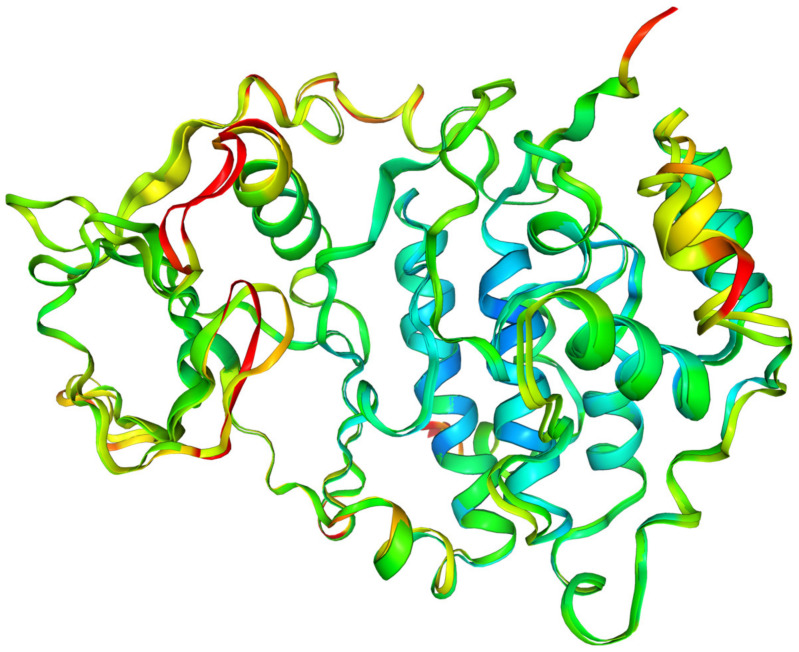
The comparison of the rigidity of 2OXY and 4KWP. The normalized B-factors (Debye–Waller factor/atomic displacement parameter) were used as a measure. The more rigid fragments are indicated in red, while the less rigid ones are indicated in blue (scale: red +4, blue −2).

A comparison of the rigidity of 2OXY and 4KWP using the normalized B-factors (Debye–Waller factor or atomic displacement parameter) is shown in Figure 12. 

It helps to identify the flexibility of the specific protein regions. 4KWP, determined with a higher accuracy than 2OXY (1.25 vs. 1.81 Å), displays greater flexibility, particularly in the region of the main hydrophobic 77 and 174 residues, which are of paramount importance for binding. The remaining residues of the active site are less flexible than the non-active site residues, which is a typical characteristic. Correlating the B-factor with binding affinity leads to the conclusion that the greater the stiffness of the complex, the higher the binding affinity. The higher binding affinity for 3KXM (−7.77 kcal/mol) and 4KWP (−7.66 kcal/mol) than 2OXY (−7.07 kcal/mol) is in agreement with the above observation.

**Figure 13:**
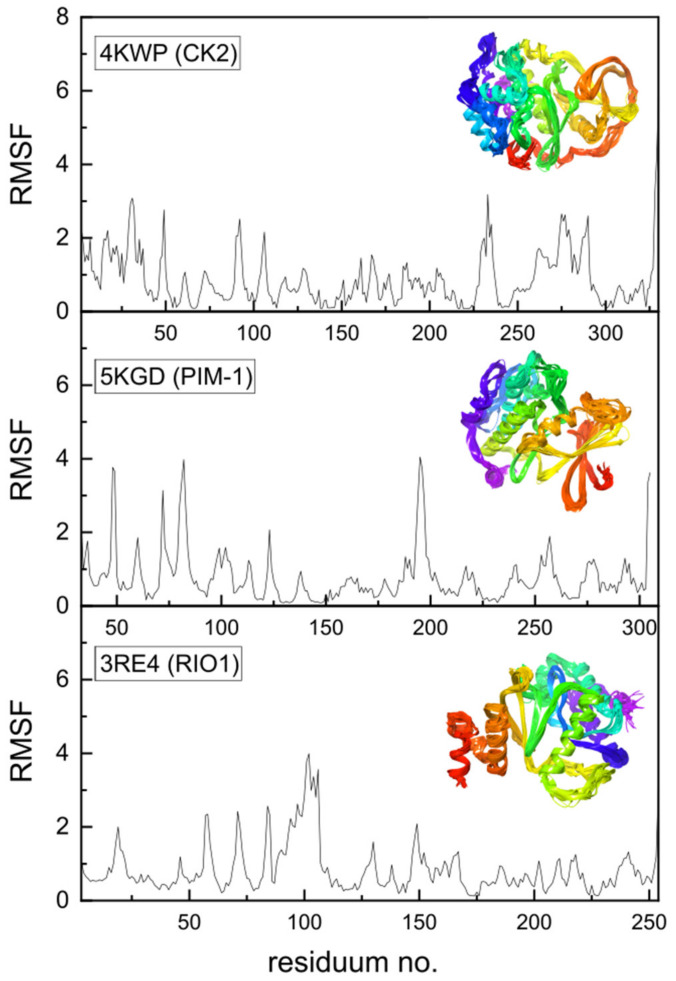
The root-mean-square fluctuations (RMSF in Å^2^) vs. residue number for 4KWP (CK2α)—top, 5KGD (PIM-1)—middle, and 3RE4 (RIO1)—bottom. The multiple models obtained using the coarse-grained approach and overlapped reveal the flexibility of the whole proteins and are shown in the inset (residue position color scheme was used).

Additional data may be provided by the Molecular Dynamics Simulation, which has been performed using a coarse-grained approach. Coarse-grained modelling aims to simulate the behavior of complex systems using a simplified representation that limits computational complexity. Higher root-mean-square fluctuation (RMSF) values for Cα atoms in amino acid residues can be treated as a measure of the fluctuations in the atomic positions and flexibility of the whole residues. Protein regions characterized by higher RMSF are more flexible, less stable, and more dynamic, whereas those with lower RMSF are more rigid and stable. High RMSF peaks indicate regions of greater flexibility, as seen in Figure 13, which may be functionally important. The protein’s flexibility was visualized using multiple models from MDS that were superimposed on each other; the effect is illustrated in the inset of Figure 13.

In the native state, i.e., under crystalline conditions, only a few side chains, 49, 92, 230, 232, 235, 264, 267, 279, and 283, and terminal 3 and 330 show increased flexibility in 4KWP. However, the RMSF, which is the time average of the root-mean-square deviation of atomic structures (RMSD) does not exceed 2.5 Å. Assuming completely free small protein chains, i.e., very high side-chain mobility, the residues 106, 92, and 231 and terminal 9 and 329 have been detected as more flexible, with the RMSF value not exceeding 3.5 and 5 Å, respectively. In standard conditions, the RMSF for 4KWP does not exceed 3.5 Å. The RMSF for 5KGD and 3RE4 in standard conditions does not exceed 4.0 Å; in crystalline conditions it is even lower and does not exceed 2.6 and 2.7 Å, respectively.

The active sites in CK2α, PIM-1, and RIO1 are characterized by a high degree of stiffness, which can be clearly seen in Figure 13. The observed variations in binding modes fall within the established limits of repeatability when employing distinct MD approaches, confirming the conclusions drawn from flexible molecular docking. The outcomes derived from the MDS are in alignment with the conclusions drawn from the analysis of the B-factors. The variations in binding modes were found insignificant and irrelevant from the point of view of active site research, falling within the range produced by the various docking techniques, and do not affect the final conclusions.

**Figure 14:**
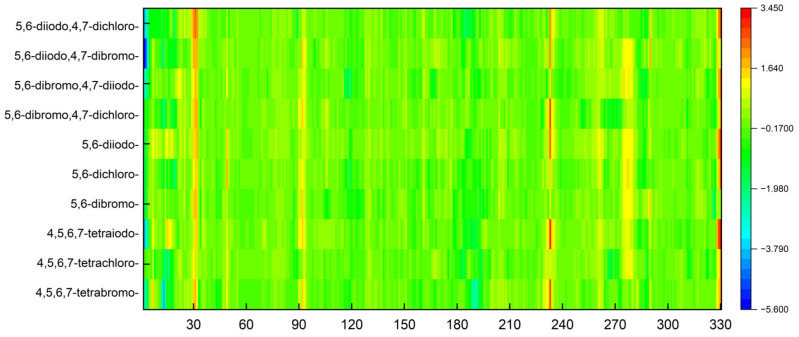
A comparison of the differences between the RMSF of different CK2α –ligand complexes in relation to 4KWP (ligand: 1-(β-D-2′-deoxyribofuranosyl)-4,5,6,7-tetrabromo-1*H*-benzimidazole).

The objective of the MDS study was also to determine the extent to which the ligands from the 2′-deoxy-2′,2′-difluoro-ribose series affect the structural rigidity of the CK2α target. The findings are presented in the form of heatmaps in Figure 14.

In the area of residues 30, 92, and 233, the stiffness of the protein–ligand structure under investigation is greater than the stiffness of the native 4KWP, as shown in Figure 14. The influence of the interactions formed by each ligand on the flexibility and stiffness of the predicted complexes is small. The decrease in rigidity is observed only in structures with 5,6-diiodo,4,7-dibromo- and 4,5,6,7-tetrabromo- as ligands and concerns residues 3 and 13, respectively. Both are located at a considerable distance from the active site. The greater the stiffness of the protein–ligand complex, the greater the binding affinity. Moreover, in all cases examined here, the stiffness provided by the protein structure appears to serve to enhance rather than oppose selectivity.

### 2.5. Structure-Binding Affinity Analysis Using Network-like Similarity Graphs

#### 2.5.1. Structure-Activity Landscape Index

The docked ligands were subjected to further structure–activity relationship analysis using Network-like Similarity Graphs [72], where the studied molecules are represented as nodes and edges are used to connect similar structures. The size of the nodes in the network similarity graphs are defined by the Structure-Activity Landscape Index (SALI) [73] as follows:

(1) $$SALI=\frac{\left|A_{1}-A_{2}\right|}{1-s}$$

and this can serve as a measure of the difference in the experimental values of the activities of A_1_ and A_2_ with respect to their structural similarity s. The SALI value represents the degree of change in activity resulting from a relatively minor structural alteration. The similarity of chemical structures can be described by Skeleton Spheres, a measure used to determine the total number of “round” matching fragments in two molecules being compared. The high degree of similarity in the 3D-shape of the molecules (or their large parts) is accompanied by a Skeleton Spheres proximity.

**Figure 15:**
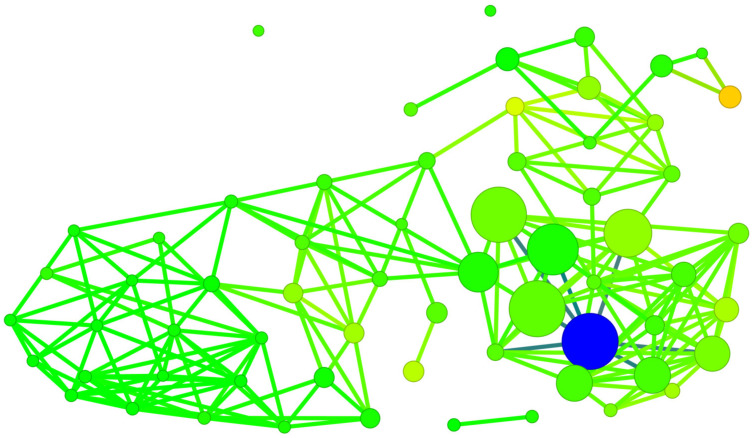
A diagram of binding efficiency versus Skeleton Spheres for polyhalogeno-1*H*-benzimidazole inhibitors for which the inhibitor constant, K_i_, has been experimentally determined. The size and color of the dots correspond to the SALI/Skeleton Spheres and K_i_ values. The most promising polyhalogeno-1*H*-benzimidazole inhibitor is depicted in blue.

The SALI diagram shown in Figure 15 facilitates the straightforward identification of activity cliffs, i.e., cases when a sudden change in activity is achieved with little structural modification. The most promising modification in polyhalogeno-1*H*-benzimidazoles indicated by SALI is substitution with –CF_3_ or –CF_2_F_3_ at C(2).

However, in the case of the polyhalogenated-1*H*-benzimidazole inhibitors, the Skeleton Spheres parameter shows large activity spikes (green circles) for ligands which have limited binding efficiency. Consequently, this similarity measure is not the optimal one.

An alternative approach is the use of the 3D pharmacophore, which is defined as an ensemble of structural features that are crucial to attach or bind to an active site of an enzyme or molecule, i.e., necessary for molecular recognition [74]. Its undoubted advantage lies in the fact that it not only incorporates the ligand’s shape, but also its entire set of features.

**Figure 16:**
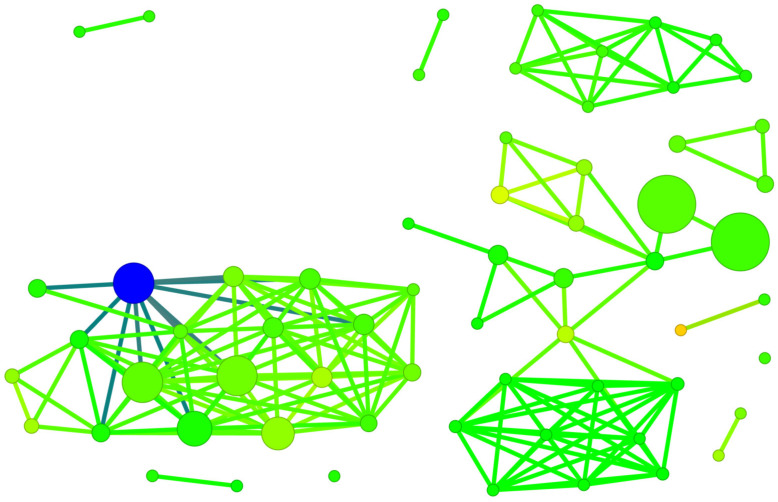
A diagram of binding efficiency versus 3D Flexophore for kinase inhibitors for which the inhibitor constant, K_i_, has been experimentally determined. The size and color of the dots correspond to the SALI/3D Flexophore and K_i_ values. The most promising polyhalogeno-1*H*-benzimidazole inhibitor is depicted in blue.

The diagram shown in Figure 16 is a bit more reliable and offers a more comprehensive illustration of the relationship between ligands.

The division into individual groups of ligands using 3D Flexophore similarity, shown in Figure 16, is more reliable than when using Skeleton Spheres for similarity, shown in Figure 15, although the activity cliffs do not differ.

#### 2.5.2. Structure-Binding Affinity Index and Structure-Binding Affinity Landscape

The analysis described above is typically performed using experimental activity parameters (e.g., inhibitor constant, K_i_; dissociation constant, k_d_; and half maximal inhibitory concentration, IC50). Nevertheless, when screening for new ligands, it is possible that the experimental data may be incomplete or absent. Therefore, we propose an alternative approach, using binding affinity, to address this issue. We defined a new parameter Structure-Binding Affinity Index, SBAI:

(2) $$SBAI=\frac{\left|{BA}_{1}-{BA}_{2}\right|}{1-s}=\frac{{|d}_{BA}|}{1-s}$$

i.e., the difference in the binding affinity of the ligands (numerator), d_BA_, with respect to the structural dissimilarity, 1 − s, (denominator), with the structural similarity, s, determined using Skeleton Spheres or the 3D Flexophore. The 3D Flexophore descriptor permits the prediction of the 3D pharmacophores’ similarity and facilitates the assessment of ligand compatibility in terms of protein binding.

**Figure 17:**
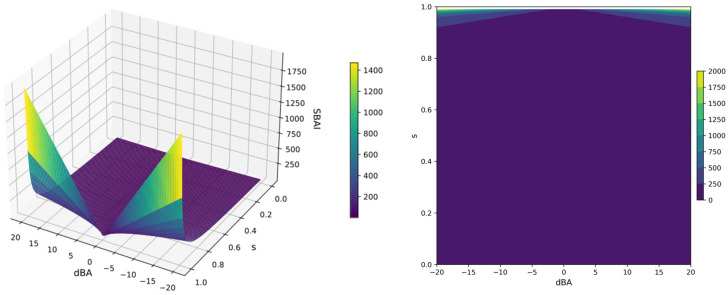
The 3D surface (**left**) and contour (**right**) plots of the Structure-Binding Affinity Index SBAI(d_BA_, s); the s-variable range from 0 to 1, the d_BA_-variable range from −20 to 20 kcal/mol and the SBAI range from 0 to infinity, truncated at 1500 (**left**) and 2000 (**right**).

The SBAI index is a measure of the extent to which binding affinity is gained or lost in response to a relatively small change in the ligand’s structure. Rapid changes in the SBAI(d_BA_,s) function in the high range of the s and d_BA_ values are clearly visible in the 3D surface and contour plots, shown in Figure 17. For visualization, the s-variable ranged from 0 to 1 and the d_BA_-variable from −20 to 20 kcal/mol and the SBAI, which reaches infinite values, has been truncated for clarity.

The smaller the structural difference between the ligands, the higher the SBAI value; for two identical ligands, SBAI(0, 1) is infinitely large, as seen in Figure 17. SBAI in fact enables the identification of binding affinity cliffs, when a sudden change in binding affinity is achieved with little structural modification. The heatmap for ligand pairs generated in this manner enables the identification of the ligands, which seem the most promising for further investigation.

**Figure 18:**
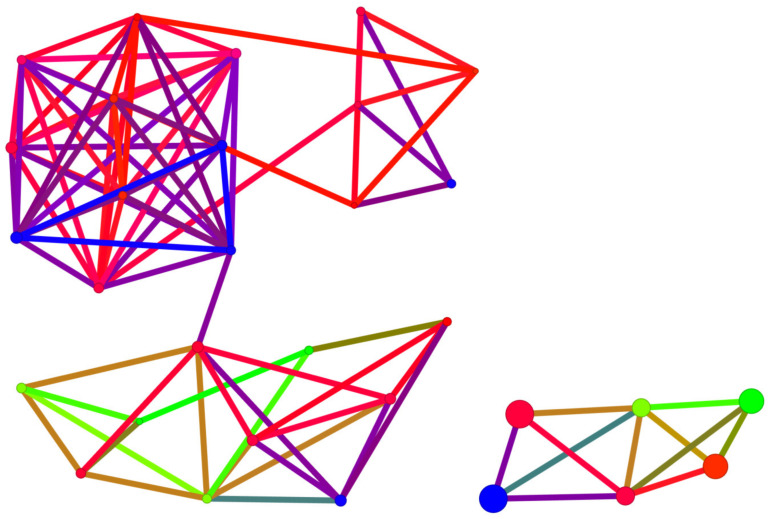
A diagram of binding affinity versus Skeleton Spheres for the studied kinase inhibitors. The 4,5,6,7-tetraiodo- and 5,6-diiodo- derivatives formed a completely separate group. The size and colour of the dots correspond to the SBAI/3D Flexophore values. The polyhalogeno-1*H*-benzimidazole ligand depicted by the large circle in blue is 4,5,6,7-tetraiodo- from the 2′-deoxy-2′,2′-difluoro-ribose series.

**Figure 19:**
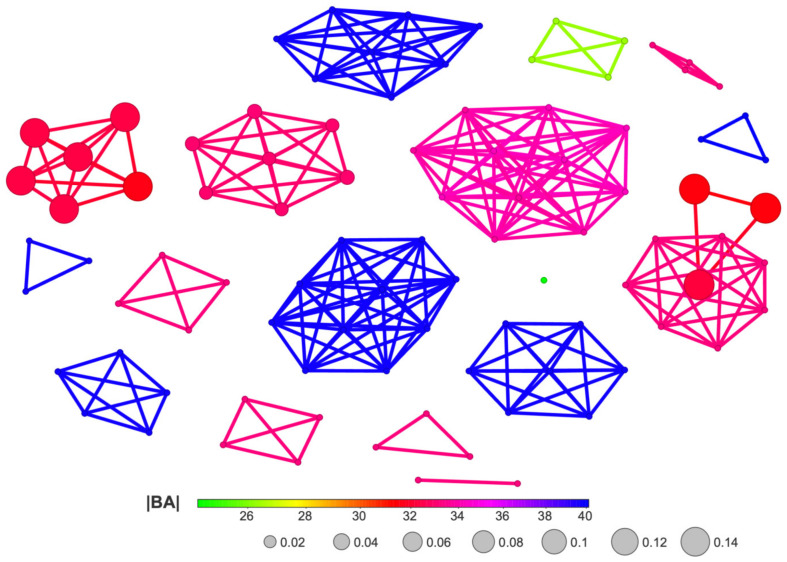
A SBAI diagram, with Skeleton Spheres as the similarity measure, for the studied ligands.

Since both Skeleton Spheres and the 3D Flexophore can be used for the calculation of the similarity parameter, we have shown both variants of SBAI in Figure 18 and Figure 19.

A structurally distinct subgroup among the studied ligands is constituted by derivatives containing only iodine atoms. Among these, 4,5,6,7-tetraiodo- ligands from the 2′-deoxy-2′,2′-difluoro-ribose series display the most distinctive structural features, as shown in Figure 18. An analysis of the SBAI diagram with Skeleton Spheres leads to the conclusion that the binding affinity is much more influenced by the aglycone than the glycone modification.

**Figure 20:**
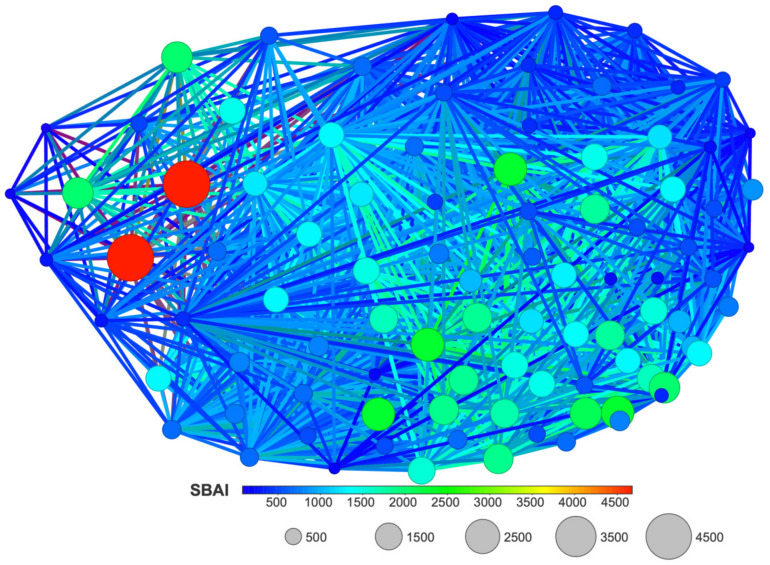
A Structure-Binding Affinity Landscape, with the 3D Flexophore as a similarity measure, for the studied ligands.

The SBAI diagram with the 3D Flexophore as a measure of the ligands’ similarity, the so-called Structure-Binding Affinity Landscape, Figure 20, has a form of binding affinity map that helps interpret the docking results in the context of notable increases/decreases in the binding affinity related to structural changes.

The largest red cliffs correspond to the 4,5,6,7-tetraiodo- and 5,6-dioodo- ligands, while the medium green ones correspond to the 5,6-diiodo-4,7-dibromo- ligand from the 2′-deoxy-2′,2′-difluoro- series. The aforementioned three compounds appear to be the most promising candidates for further investigation. These findings are in good accordance with the results obtained from MD and MDS. Moreover, the analysis of the already known ligands, Figure S1, supported our method and the finding that fluorine modification is highly promising.

Our method for quantifying differences between the ligands and their binding capabilities as well as screening them using the Structure-Binding Affinity Index and Structure-Binding Affinity Landscape holds promise for guiding future research on new anti-cancer agents. The aforementioned approach may prove advantageous in the design of other drugs targeting a number of diseases, an example of which we will show in our forthcoming paper [75].

## 3. Materials and Methods

### 3.1. ADME and Membrane Permeability Prediction

The in silico ADME (absorption, distribution, metabolism, and excretion) drug-likeness evaluation was performed using the SwissADME tool developed by the Swiss Institute of Bioinformatics (Lausanne, Switzerland) [60]. Drug-likeness was tested according to the Lipinski, Veber, and Egan rules of 5 (RO5). The Abbot bioavailability scores were computed to predict the probability of a compound to have at least 10% oral bioavailability. Lipophilicity was predicted with iLOGP, XLOGP3, WLOGP, MLOGP, and SILICOS-IT models from which a consensus log Po/w was determined [60]. The solubility (log S) of the ligands was predicted using SILICOS-IT [60]. The synthetic availability (SA) was determined based on the frequency of molecular fragments in “truly” obtainable molecules.

The mutagenicity/carcinogenicity risk scores, Caco-2, and Madin-Darby Canine Kidney (MDCK) intestinal cell permeability and QED, a complex measure of attractiveness calculated on the basis of drug-likeness parameters: MW, log P, NHBA, NHBD, PSA, N_rotb_, the number of aromatic rings (N_Ar_), and the number of alerts for undesirable functional groups, were predicted using models implemented in ADMET2.0 [61].

Since most drugs are absorbed passively, Caco-2 and Madin-Darby Canine Kidney (MDCK) models are widely used for assessing intestinal permeability. Recently, a new model based on the graph convolutional neural network approach was developed by the National Center for Advancing Translational Sciences (NCATS) to predict Parallel Artificial Membrane Permeability Assay (PAMPA) results in three variants for pH = 7.4 and pH = 5.0 and the blood–brain barrier (BBB) [62]. PAMPA determines the permeability of substances from a donor compartment, through a lipid-infused artificial membrane, and into an acceptor compartment.

### 3.2. Density Functional Theory

The quantum chemical calculations required for the QSPR and QTAIM analysis and subsequent MD study were carried out within the density functional theory (DFT). The hybrid meta exchange-correlation functional, with a double amount of non-local exchange, M062X, which provides a reliable electron density distribution in a molecular system with non-covalent internal interactions [58], combined with an all-electron split-valence basis set 6-311+G(d,p), was used. M062X seems the optimal choice from the point of view of accuracy and algorithmic complexity. All of the calculations were carried out using Gaussian 16 rev. C01 [76].

### 3.3. Molecular Docking

The molecular docking (MD) approach is frequently used to model protein–ligand interactions because it identifies binding sites to the target protein and optimizes the ligand structure. Thus, it is an essential approach for predicting ligand activity, and determining protein–ligand binding patterns [58,59,77]. To acquire convincing findings, both the ligand structure and the protein’s reliable 3D crystallographic structure must be known. Three-dimensional (3D) molecular structures of the ligands used in this study have been optimized using Gaussian 16, rev. C01 [76], at the M062X/6-31+G(d,p) level of theory. Structures of the targets were retrieved from the Protein Data Bank (PDB) database (http://www.rcsb.org/pdb, accessed on 29 March 2024).

The molecular docking of receptor and ligand structures (previously converted to .pdbqt format, MGLTools ver. 1.5.7) was performed using AutoDock ver. 4.2.6 [78] and AutoDock Vina ver. 1.2.3 [79]. The native ligands that co-crystallized with CK2α and water were removed before docking. The protonation state of the protein itself was corrected before docking. In order to refine the docking protocol, a test redocking of the original ligand was performed, and the result was evaluated. The protocol was considered satisfactory when the pose’s root-mean-square deviation (RMSD) from its conformation in the parent structure met the criterion of RMSD < 3 Å. A weakness of classical molecular docking is the lack of the flexibility of the residues. Therefore, the docking results were verified using two different techniques, flexible ligands and flexible residues (to the extent possible to declare). The ligands studied were docked into the protein structure using two separate techniques: template docking and docking with a defined search space (the grid box of size 9–13 Å centered on the active site). The docking results were analyzed and the optimal poses that resulted in the greatest stabilization of the protein–ligand complex were selected for further studies. The molecular docking procedure was repeated using the Genetic Evolutionary Method for molecular DOCKing (GEMDOCK) [80]. Although this technique also employs a genetic algorithm, the evolution operator crucial for the navigation in the search space is distinct. This plays an important role in terms of constraints on moving around the different hyperplanes of the domain and, consequently, the success of the optimization algorithm. The interaction energy was the sum of the contributions of the piecewise linear potential (PLP) (steric, van der Waals and hydrogen bonds) and Coulomb (electrostatic interactions) terms. The binding affinity was estimated using the Gehlhaar model [81] with original parameterization and using PRODIGY [82,83]. The final 2D and 3D visualizations of the binding modes were made using PoseEdit [84] and VMD [85].

### 3.4. Molecular Dynamic Simulations

The molecular dynamics simulations have been performed using coarse grain technique [82,83]. The idea of coarse-graining is to reduce computational complexity by replacing the real particles with a smaller number of representative elements whose behavior is equivalent to the original ones and whose motion can be easily tracked during the simulation. The total number of generated models was 50,000, of which 1000 were selected and the top 10 were subject to further analysis. The root-mean-square fluctuation (RMSF), defined as the time average of the root-mean-square deviation (RMSD) of atomic positions, was analyzed with regard to the conformational flexibility of individual regions. The alterations in the radius of gyration that correspond to the data regarding conformational stability were calculated.

### 3.5. Evaluation of the Binding Modes

#### 3.5.1. Root-Mean-Square Deviation of the Binding Mode

The average deviation between the binding modes was calculated using the newly defined quantity: the root-mean-square deviation of the binding modes (RMSD_BM) [58]. It was calculated as follows:

(3) $$RMSD\_BM(P,Q)=\sqrt{\frac{1}{n}\sum_{i}{\left(p_{i}-q_{i}\right)}^{2}}$$

where p_i_ and q_i_ are the binding interactions in each structure and P = {p_i_} and Q = {q_i_}.

#### 3.5.2. Heatmaps

Heat maps are a two-dimensional data visualization technique in which the magnitude of individual values in a dataset are color-coded, which helps in capturing the most relevant data. In the biological or geographical fields, heat maps are used to visually represent patterns in DNA, RNA, or gene expression, or the geographic distribution of data, respectively.

In this paper, we applied gridded color-coded heat maps to visualize:

- The binding modes of protein–ligands (a technique successfully applied in our previous papers [58,77]);
- The protein–ligand binding energy;
- The normalized B-factors;
- The root-mean-square fluctuation (RMSF) of a structure.

Native and docking-derived complexes were compared using a red–yellow–blue scheme, with dark red and dark blue indicating strong and weak interactions, respectively.

## 4. Conclusions

According to the Similar Property Principle, structurally similar molecules usually have similar physicochemical properties. This elementary principle provided the impetus for the development of an original methodology, which is presented in this paper. We designed and investigated small structural modifications of polyhalogeno-1*H*-benzimidazoles. The ADME/PAMPA analysis indicated that the designed ligands deserved further consideration. We found that a small structural change, a change in the type and/or number of halogen atoms in the aglycone moiety, or a substitution at the 2′ position of the glycone of polyhalogen-1*H*-benzimidazoles, leads to a significant change in the protein–ligand binding. MD provided the models for the optimal orientation of ligand relative to target in the formation of the stable complexes and, supplemented with the root-mean-square binding mode deviation, RMSD_BM, gave insight into the nature of the protein–ligand bindings. The mathematical metrics helped assess the global distance between the binding modes of individual ligands with their target, while heatmaps were highly effective in identifying the strongest components of the binding as well as obstacles in ligands and helped to reveal locations in the ligand that should be modified according to binding site requirements. The MDS supplemented with RMSF heatmaps facilitated the identification of residues showing increased flexibility and permitted an investigation of their stability.

**Figure 21:**
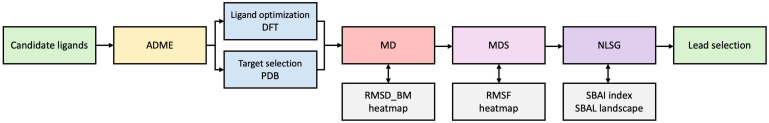
The flowchart illustrating data flows.

Nevertheless, it should be noted that none of these steps represent a straightforward process when it comes to interpreting the results. Therefore, we have proposed a novel combined approach, based on the binding modes, heatmaps, and network-like similarity graphs, which is designed to facilitate the assessment of the impact of structural changes on binding affinity. The workflow is shown in the following diagram, Figure 21.

The Structure-Binding Affinity Index and Structure-Binding Affinity Landscape proposed in this paper help to measure the extent to which binding affinity is gained or lost in response to a relatively small change in the ligand’s structure. Validation was performed using a set of 64 well-characterized biologically active kinases (polyhalogeno-1*H*-benzimidazoles), and the approach was subsequently applied to 40 designed kinases. The combined ADME/PAMPA, molecular docking, molecular dynamics simulations, and SBAI/SBAL approach facilitates the differentiation between potential drug design pathways with greater potential for success and those with limited potential. Both the SBAI and SBAL analysis of known ligands and those proposed by us suggest that an important modification may be the introduction of fluorine and it would be beneficial to investigate the potential of ligands in this direction.

Our method for quantifying differences between the ligands and their binding capabilities as well as screening them with SBAI (i.e., using a combination of binding affinity and 3D pharmacophore similarity in a single index), holds promise for guiding future research on new anti-cancer agents. The aforementioned approach can be beneficial in the design of other drugs targeted to different diseases.

## Figures and Tables

**Table 1:** Druggability and size of the cavities predicted by CavityPlus.

Target	Structure	Predicted Max pK_d_ Ligandability	Predicted Average pK_d_	DrugScore (The Degree of Druggability)	Druggability	Surface Area [Å^2^]	Volume [Å^3^]	Pharmacophore
CK2α	4KWP	11.59	6.90	785.00	Strong	835.50	1309.25	Hydrophobic centers 7 H-Bond acceptor center 1
		10.30	6.15	−31.00	Medium	718.50	944.88	-
		8.55	5.55	−1022.00	Weak	288.75	414.62	-
	8AEC	9.40	5.84	682.00	Strong	554.75	543.62	Hydrophobic centers 9
		7.48	5.18	−1204.00	Weak	222.00	291.75	-
PIM-1	4DTK	11.42	6.53	1150.00	Strong	683.75	871.62	H-Bond donor center 6 H-Bond acceptor center 3 Hydrophobic center 2
		11.06	6.41	182.00	Medium	166.00	106.38	-
	5KGD	10.77	6.13	1155.00	Strong	639.50	785.88	H-Bond acceptor center 1 Hydrophobic center 9
		9.85	6.00	−779.00	Weak	271.00	408.88	-
		8.34	5.48	−682.00	Weak	377.50	450.00	-
RIO1	3RE4	9.93	6.02	514.00	Medium	610.50	918.25	H-Bond donor center 6 H-Bond acceptor center 5 Hydrophobic center 2
		6.51	4.85	−1315.00	Weak	-	-	-
	Optimal	>6	>6	Strong: ≥600; 600 > Medium > −180	Strong/Medium	-	-	-

**Table 2:** The docking results for studied ligands (the CK2α target from 4KWP). The energy of protein–ligand, steric, van der Waals and hydrogen bond interactions is expressed in kcal/mol.

Glycone	Ligand	E_protein–ligand_	E_total_	Steric	van der Waals	Hydrogen Bond	Binding Affinity
-	4,5,6,7-tetrabromo-	−66.69	−67.37	−66.69	−15.54	0	−8.67
	4,5,6,7-tetrachloro-	−68.56	−66.16	−68.56	−15.93	0	−8.67
	4,5,6,7-tetraiodo-	−64.90	−66.47	−64.90	−14.95	0	−8.67
	5,6-dibromo-	−71.53	−69.37	−69.68	−19.98	−1.85	−7.16
	5,6-dichloro-	−72.38	−69.70	−70.48	−20.28	−1.90	−7.17
	5,6-diiodo-	−70.93	−68.88	−69.28	−19.37	−1.65	−7.14
	5,6-dibromo,4,7-dichloro-	−66.79	−66.43	−66.79	−15.60	0	−8.61
	5,6-dibromo,4,7-diiodo-	−65.27	−67.01	−65.27	−15.04	0	−8.61
	5,6-diiodo,4,7-dibromo-	−64.88	−66.72	−64.88	−14.94	0	−8.60
	5,6-diiodo,4,7-dichloro-	−64.76	−65.50	−64.76	−14.85	0	−8.61
ribose	4,5,6,7-tetrabromo-	−81.88	−82.32	−78.84	−18.54	−3.04	−7.56
	4,5,6,7-tetrachloro-	−83.69	−80.81	−80.61	−18.69	−3.08	−7.56
	4,5,6,7-tetraiodo-	−79.43	−81.02	−76.59	−18.13	−2.83	−7.54
	5,6-dibromo-	−72.79	−69.69	−70.11	−21.02	−2.68	−5.83
	5,6-dichloro-	−73.40	−69.78	−70.69	−20.92	−2.70	−5.83
	5,6-diiodo-	−73.09	−70.32	−70.59	−22.38	−2.50	−5.81
	5,6-dibromo,4,7-dichloro-	−85.28	−83.80	−80.40	−18.20	−4.88	−7.72
	5,6-dibromo,4,7-diiodo-	−80.70	−82.01	−75.94	−17.49	−4.76	−7.71
	5,6-diiodo,4,7-dibromo-	−81.15	−82.32	−77.76	−17.24	−3.39	−7.55
	5,6-diiodo,4,7-dichloro-	−84.41	−84.04	−79.54	−16.90	−4.86	−7.72
2′-deoxyribose	4,5,6,7-tetrabromo-	−83.33	−81.57	−80.83	−22.47	−2.50	−8.06
	4,5,6,7-tetrachloro-	−84.93	−86.28	−82.43	−22.55	−2.50	−7.94
	4,5,6,7-tetraiodo-	−79.78	−85.01	−77.28	−22.45	−2.50	−7.94
	5,6-dibromo-	−71.57	−71.27	−69.07	−22.34	−2.50	−6.29
	5,6-dichloro-	−72.54	−71.98	−70.04	−22.40	−2.50	−6.29
	5,6-diiodo-	−71.09	−70.96	−68.59	−22.48	−2.50	−6.29
	5,6-dibromo,4,7-dichloro-	−84.10	−87.25	−81.60	−22.47	−2.50	−7.94
	5,6-dibromo,4,7-diiodo-	−81.00	−86.09	−78.50	−22.67	−2.50	−7.94
	5,6-diiodo,4,7-dibromo-	−81.82	−86.77	−79.32	−22.48	−2.50	−7.94
	5,6-diiodo,4,7-dichloro-	−83.07	−87.08	−80.57	−22.46	−2.50	−7.94
2′-deoxy- 2′,2′-difluoro-ribose	4,5,6,7-tetrabromo-	−77.05	−61.06	−75.30	−22.63	−1.76	−9.53
	4,5,6,7-tetrachloro-	−77.84	−60.06	−76.16	−22.65	−1.69	−9.52
	4,5,6,7-tetraiodo-	−77.73	−63.48	−75.38	−22.87	−2.35	−9.59
	5,6-dibromo-	−65.83	−44.21	−64.10	−22.71	−1.73	−7.89
	5,6-dichloro-	−67.34	−44.52	−65.58	−22.97	−1.76	−7.89
	5,6-diiodo-	−66.60	−44.22	−64.62	−22.81	−1.98	−7.92
	5,6-dibromo,4,7-dichloro-	−77.45	−61.53	−75.75	−22.62	−1.70	−9.52
	5,6-dibromo,4,7-diiodo-	−74.65	−60.67	−72.94	−22.68	−1.70	−9.52
	5,6-diiodo,4,7-dibromo-	−76.62	−62.32	−74.88	−22.47	−1.74	−9.53
	5,6-diiodo,4,7-dichloro-	−76.60	−61.45	−74.92	−22.57	−1.68	−9.52

**Table 3:** The docking results for studied ligands from the 2′-deoxy-2′,2′-difluoro-ribose series (the PIM-1 target from 5KGD). The energy of protein–ligand, steric, van der Waals and hydrogen bond interactions is expressed in kcal/mol.

Ligand	E_protein–ligand_	E_total_	Steric	van der Waals	Hydrogen Bonds	Binding Affinity
4,5,6,7-tetrabromo-	−91.79	−77.06	−87.79	−15.63	−4.00	−9.80
4,5,6,7-tetrachloro-	−96.61	−79.36	−92.83	−19.63	−3.79	−9.78
4,5,6,7-tetraiodo-	−83.33	−70.88	−79.35	−21.12	−3.98	−9.79
5,6-dibromo-	−94.13	−81.73	−89.13	−18.71	−5.00	−8.29
5,6-dichloro-	−96.82	−83.81	−91.82	−18.87	−5.00	−8.29
5,6-diiodo-	−90.29	−78.04	−85.29	−18.55	−5.00	−8.29
5,6-dibromo,4,7-dichloro-	−91.90	−76.44	−87.97	−20.62	−3.93	−9.79
5,6-dibromo,4,7-diiodo-	−88.96	−76.35	−84.99	−21.96	−3.97	−9.79
5,6-diiodo,4,7-dibromo-	−85.14	−71.34	−81.18	−20.38	−3.96	−9.79
5,6-diiodo,4,7-dichloro-	−86.87	−72.25	−82.89	−20.36	−3.97	−9.80

**Table 4:** The docking results for studied ligands from the 2′-deoxy-2′,2′-difluoro-ribose series (the RIO1 target from 3RE4). The energy of protein–ligand, steric, van der Waals and hydrogen bond interactions is expressed in kcal/mol.

Ligand	E_protein–ligand_	E_total_	Steric	van der Waals	Hydrogen Bond	Binding Affinity
4,5,6,7-tetrabromo-	−81.75	−60.95	−80.65	−24.66	−1.09	−9.45
4,5,6,7-tetrachloro-	−86.01	−64.41	−85.09	−23.19	−0.93	−9.44
4,5,6,7-tetraiodo-	−75.61	−57.06	−75.61	−25.47	0	−9.32
5,6-dibromo-	−81.87	−71.31	−79.97	−22.28	−1.90	−7.91
5,6-dichloro-	−84.21	−73.30	−82.23	−21.75	−1.99	−7.92
5,6-diiodo-	−79.76	−69.26	−77.74	−21.75	−2.03	−7.93
5,6-dibromo,4,7-dichloro-	−83.77	−64.10	−82.95	−22.97	−0.82	−9.43
5,6-dibromo,4,7-diiodo-	−80.14	−61.61	−80.14	−27.26	0	−9.32
5,6-diiodo,4,7-dibromo-	−79.29	−60.33	−78.86	−24.06	−0.43	−9.38
5,6-diiodo,4,7-dichloro-	−81.66	−62.87	−80.77	−22.98	−0.90	−9.43

## Data Availability

All data are included in the text of the manuscript.

## Supplementary Materials

The following supporting information can be downloaded at: https://www.mdpi.com/article/10.3390/molecules29133199/s1, Table S1. The physicochemical profile parameters that describe the pharmacokinetic behavior of the studied ligands; Table S2. The parameters describing the toxicity of the studied ligands; Table S3. The permeability profile parameters for the studied ligands; Table S4. Comparison of the binding modes of the individual ligands with respect to reference ligand the reference ligand; Table S5. Comparison of the binding modes of the individual ligands with respect to the native ligand (atomic terms); Figure S1. A SBAI diagram, with Skeleton Spheres as similarity measure, for the large set of studied ligands (validation task).
