# Supplementary material for: Serine/Threonine Protein Kinases as Attractive Targets for Anti-Cancer Drugs—An Innovative Approach to Ligand Tuning Using Combined Quantum Chemical Calculations, Molecular Docking, Molecular Dynamic Simulations, and Network-like Similarity Graphs
Source: Molecules. 2024 Jul 5;29(13):3199. doi: 10.3390/molecules29133199 (PMC11243552; doi:10.3390/molecules29133199)
Supplement: Supplementary file 1 [file molecules-29-03199-s001.zip › molecules-3062784-supplementary.pdf]

molecules

MDPI

Supplementary

Serine/Threonine Protein Kinases as Attractive Targets for Anti-Cancer Drugs—An Innovative Approach to Ligand Tuning Using Combined Quantum Chemical Calculations, Molecular Docking, Molecular Dynamic Simulations, and Network-like Similarity Graphs

Magdalena Latosińska and Jolanta Natalia Latosińska

Table S1. The physicochemical profile parameters that describe the pharmacokinetic behavior of the studied ligands.

glycone	Ligand	MW	QED	Golden triangle	LogP	Consensus LogP	Solubility (SILICOS-IT)	TPSA [A²]	SAS	CaCo-2	MDCK	BBB
	4,5,6,7-tetrabromo-	435.74	0.62	Yes	3.42	2.66	-4.46	24.39	4.28	-4.849	1.2142e-05	0.84
	4,5,6,7-tetrachloro-	257.93	0.709	Yes	3.27	2.26	-3.63	24.39	4.30	-4.742	9.1739e-06	0.464
	4,5,6,7-tetraiodo-	623.74	0.411	No	4.57	2.85	-4.63	24.39	4.13	-4.536	1.5762e-05	0.597
	5,6-dibromo-	277.94	0.719	Yes	2.13	1.69	-2.43	24.39	4.23	-4.917	1.1871e-05	0.88
	5,6-dichloro-	189.04	0.615	No	2.06	1.49	-1.96	24.39	4.20	-4.752	1.3415e-05	0.726
-	5,6-diiodo-	371.94	0.647	Yes	2.71	1.80	-2.57	24.39	4.14	-4.558	1.1356e-05	0.507
	5,6-dibromo,4,7-dichloro-	346.83	0.716	Yes	3.34	2.47	-4.06	24.39	4.32	-4.706	1.0427e-05	0.459
	5,6-dibromo,4,7-diiodo-	529.74	0.478	No	4.00	2.74	-4.55	24.39	4.21	-4.844	1.4083e-05	0.442
	5,6-diiodo,4,7-dibromo-	529.74	0.478	No	4.00	2.77	-4.55	24.39	4.25	-4.602	1.2736e-05	0.685
	5,6-diiodo,4,7-dichloro-	440.84	0.575	Yes	3.92	2.57	-4.18	24.39	4.24	-4.628	1.3498e-05	0.626
	4,5,6,7-tetrabromo-	565.83	0.385	No	2.10	2.28	-3.84	87.74	3.93	-5.131	1.5947e-05	0.627
	4,5,6,7-tetrachloro-	388.03	0.543	Yes	1.95	1.93	-3.09	87.74	3.77	-5.002	1.4356e-05	0.082
	4,5,6,7-tetraiodo-	753.84	0.249	No	3.25	2.44	-3.97	87.74	4.06	-4.976	1.7369e-05	0.252
	5,6-dibromo-	410.06	0.601	Yes	-0.06	0.28	-0.24	85.52	5.24	-5.181	1.8319e-05	0.196
ribose	5,6-dichloro-	321.16	0.654	Yes	-0.13	0.10	0.17	85.52	5.20	-5.116	4.8075e-05	0.448
	5,6-diiodo-	504.06	0.467	No	0.52	0.40	-0.34	85.52	5.17	-5.098	2.0764e-05	0.776
	5,6-dibromo,4,7-dichloro-	476.93	0.458	Yes	2.02	2.08	-3.48	87.74	3.85	-5.065	1.1498e-05	0.232
	5,6-dibromo,4,7-diiodo-	661.85	0.392	No	1.85	1.32	-2.27	85.52	5.24	-5.132	1.5637e-05	0.259
	5,6-diiodo,4,7-dibromo-	659.84	0.262	No	2.68	2.40	-3.91	87.74	4.03	-5.065	1.1537e-05	0.174
	5,6-diiodo,4,7-dichloro-	570.93	0.293	No	2.60	2.19	-3.56	87.74	3.90	-4.949	1.5533e-05	0.158
	4,5,6,7-tetrabromo-	549.84	0.443	No	2.98	3.11	-4.67	67.51	3.68	-4.99	1.53687e-05	0.554
	4,5,6,7-tetrachloro-	372.03	0.627	Yes	2.83	2.73	-3.91	67.51	3.51	-4.837	2.2527e-05	0.119
	4,5,6,7-tetraiodo-	737.84	0.283	No	4.14	3.27	-4.80	67.51	3.82	-4.779	2.2073e-05	0.113
	5,6-dibromo-	392.04	0.823	Yes	1.62	1.87	-3.13	67.51	3.52	-4.821	1.67186e-05	0.166
2′-deoxyribose	5,6-dichloro-	303.14	0.89	Yes	1.55	1.70	-2.72	67.51	3.43	-4.647	1.5554e-05	0.217
	5,6-diiodo-	486.04	0.637	Yes	2.20	1.95	-3.24	67.51	3.62	-4.548	1.4471e-05	0.475
	5,6-dibromo,4,7-dichloro-	460.93	0.531	Yes	2.91	2.96	-4.30	67.51	3.60	-4.914	1.5226e-05	0.149
	5,6-dibromo,4,7-diiodo-	643.84	0.299	No	3.56	3.18	-4.74	67.51	3.92	-4.904	1.5192e-05	0.31
	5,6-diiodo,4,7-dibromo-	643.84	0.299	No	3.56	3.21	-4.74	67.51	3.80	-4.926	1.5113e-05	0.264
	5,6-diiodo,4,7-dichloro-	554.93	0.34	No	3.48	3.01	-4.39	67.51	3.66	-4.766	1.9712e-05	0.161
	4,5,6,7-tetrabromo-	585.82	0.414	No	3.41	3.50	-5.17	67.51	3.80	-5.013	1.5840e-05	0.691
	4,5,6,7-tetrachloro-	408.01	0.59	Yes	3.27	3.15	-4.42	67.51	3.68	-4.859	2.6835e-05	0.172
	4,5,6,7-tetraiodo-	773.82	0.28	No	4.57	3.66	-5.29	67.51	3.92	-4.843	2.5939e-05	0.053
2′-deoxy-2′,2′-difluoro-ribose	5,6-dibromo-	428.02	0.772	Yes	2.03	2.28	-3.65	67.51	3.68	-4.841	1.9601e-05	0.213
	5,6-dichloro-	339.12	0.881	Yes	1.96	2.15	-3.25	67.51	3.60	-4.667	2.2889e-05	0.189
	5,6-diiodo-	522.03	0.594	No	2.61	2.37	-3.74	67.51	3.78	-4.561	2.2695e-05	0.197
	5,6-dibromo,4,7-dichloro-	496.91	0.491	Yes	3.34	3.37	-4.81	67.51	3.75	-4.944	1.7415e-05	0.297
	5,6-dibromo,4,7-diiodo-	679.82	0.289	No	3.99	3.55	-5.24	67.51	3.99	-4.946	1.7997e-05	0.223
	5,6-diiodo,4,7-dibromo-	681.83	0.441	No	3.17	2.53	-3.60	65.29	5.19	-4.993	1.5992e-05	0.364
	5,6-diiodo,4,7-dichloro-	590.92	0.32	No	3.92	3.39	-4.89	67.51	3.82	-4.821	2.5532e-05	0.135
	Optimal values	**<500**	**>0.67**	**200<MW<5 -2<logD<5**	**1-4**	**1-3**	**>0**	**<140**	**-**	**>-5.15**	**>20E-06**	**1 for BBB+**

Table S2. The parameters describing the toxicity of the studied ligands.

glycone	Ligand	PAINS	BRENK	PGP sub-strate	PPB [%]	Carcinogen-icity	Genotoxic carciono-genicty /mu-tagenicty	AMES	H-HT	DILI
-	4,5,6,7-tetrabromo-	No	0	No	91.18	0.617	0	0.02	0.478	0.793
	4,5,6,7-tetrachloro-	No	0	No	27.30	0.258	0	0.023	0.843	0.494
	4,5,6,7-tetraiodo-	No	1	No	63.46	0.204	0	0.01	0.116	0.116
	5,6-dibromo-	No	0	No	50.04	0.942	0	0.926	0.793	0.038
	5,6-dichloro-	No	0	No	26.54	0.919	0	0.88	0.855	0.033
	5,6-diiodo-	No	1	No	22.04	0.711	0	0.018	0.569	0.014
	5,6-dibromo,4,7-dichloro-	No	0	No	64.76	0.692	0	0.05	0.647	0.619
	5,6-dibromo,4,7-diiodo-	No	1	No	64.46	0.611	0	0.03	0.284	0.321
	5,6-diiodo,4,7-dibromo-	No	1	No	73.95	0.248	0	0.009	0.153	0.307
	5,6-diiodo,4,7-dichloro-	No	1	No	91.19	0.262	0	0.016	0.187	0.148
ribose	4,5,6,7-tetrabromo-	No	2	Yes	98.48	0.24	0	0.153	0.199	0.957
	4,5,6,7-tetrachloro-	No	2	Yes	97.75	0.498	0	0.021	0.19	0.945
	4,5,6,7-tetraiodo-	No	3	Yes	66.01	0.039	0	0.094	0.196	0.384
	5,6-dibromo-	No	0	Yes	43.79	0.913	0	0.191	0.451	0.045
	5,6-dichloro-	No	0	Yes	21.88	0.899	0	0.132	0.467	0.027
	5,6-diiodo-	No	1	Yes	16.68	0.693	0	0.015	0.247	0.029
	5,6-dibromo,4,7-dichloro-	No	2	Yes	97.78	0.581	0	0.039	0.213	0.954
	5,6-dibromo,4,7-diiodo-	No	1	Yes	58.72	0.552	0	0.063	0.138	0.483
	5,6-diiodo,4,7-dibromo-	No	3	Yes	93.45	0.112	0	0.125	0.207	0.816
	5,6-diiodo,4,7-dichloro-	No	3	Yes	83.50	0.2	0	0.044	0.204	0.749
	4,5,6,7-tetrabromo-	No	2	Yes	98.50	0.171	0	0.044	0.229	0.97
	4,5,6,7-tetrachloro-	No	2	Yes	97.63	0.439	0	0.014	0.265	0.949
	4,5,6,7-tetraiodo-	No	3	No	65.49	0.038	0	0.03	0.268	0.615
	5,6-dibromo-	No	0	Yes	94.08	0.615	0	0.05	0.29	0.943
	5,6-dichloro-	No	0	No	80.50	0.699	0	0.03	0.486	0.933
	5,6-diiodo-	No	1	No	70.69	0.083	0	0.027	0.305	0.802
	5,6-dibromo,4,7-dichloro-	No	2	Yes	97.81	0.45	0	0.017	0.286	0.964
	5,6-dibromo,4,7-diiodo-	No	3	Yes	92.05	0.084	0	0.031	0.242	0.895
	5,6-diiodo,4,7-dibromo-	No	3	Yes	94.26	0.094	0	0.035	0.245	0.89
	5,6-diiodo,4,7-dichloro-	No	3	Yes	83.96	0.164	0	0.019	0.285	0.848
2′-de-oxy-2′,2′-difluoro-ribose	4,5,6,7-tetrabromo-	No	2	Yes	99.14	0.53	1	0.038	0.357	0.978
	4,5,6,7-tetrachloro-	No	2	Yes	98.34	0.848	1	0.009	0.822	0.973
	4,5,6,7-tetraiodo-	No	3	No	71.20	0.132	1	0.017	0.407	0.875
	5,6-dibromo-	No	0	Yes	95.26	0.861	1	0.13	0.755	0.972
	5,6-dichloro-	No	0	Yes	88.98	0.864	1	0.096	0.886	0.968
	5,6-diiodo-	No	1	Yes	76.42	0.524	1	0.038	0.589	0.953
	5,6-dibromo,4,7-dichloro-	No	2	Yes	98.39	0.794	1	0.02	0.648	0.977
	5,6-dibromo,4,7-diiodo-	No	3	Yes	94.77	0.291	1	0.022	0.372	0.963
	5,6-diiodo,4,7-dibromo-	No	1	Yes	75.07	0.613	1	0.012	0.198	0.805
	5,6-diiodo,4,7-dichloro-	No	3	Yes	88.60	0.621	1	0.011	0.65	0.958
	Optimal values	No	0	No	<90	0	0	0	0	0

Table S3. The permeability profile parameters for the studied ligands.

glycone	Ligand	PAMPA (pH=7.4)	PAMPA50 (pH=5.0)	PAMPABBB
-	4,5,6,7-tetrabromo-	low or moderate	moderate or high	moderate or high
	4,5,6,7-tetrachloro-	high	moderate or high	moderate or high
	4,5,6,7-tetraiodo-	low or moderate	moderate or high	moderate or high
	5,6-dibromo-	low or moderate	moderate or high	moderate or high
	5,6-dichloro-	high	moderate or high	moderate or high
	5,6-diiodo-	low or moderate	moderate or high	moderate or high
	5,6-dibromo,4,7-dichloro-	high	moderate or high	moderate or high
	5,6-dibromo,4,7-diiodo-	low or moderate	moderate or high	moderate or high
	5,6-diiodo,4,7-dibromo-	low or moderate	moderate or high	moderate or high
	5,6-diiodo,4,7-dichloro-	low or moderate	moderate or high	moderate or high
ribose	4,5,6,7-tetrabromo-	low or moderate	moderate or high	low
	4,5,6,7-tetrachloro-	low or moderate	moderate or high	moderate or high
	4,5,6,7-tetraiodo-	high	moderate or high	moderate or high
	5,6-dibromo-	low or moderate	moderate or high	low
	5,6-dichloro-	low or moderate	low	low
	5,6-diiodo-	low or moderate	moderate or high	low
	5,6-dibromo,4,7-dichloro-	low or moderate	moderate or high	low
	5,6-dibromo,4,7-diiodo-	low or moderate	moderate or high	moderate or high
	5,6-diiodo,4,7-dibromo-	high	moderate or high	moderate or high
	5,6-diiodo,4,7-dichloro-	high	moderate or high	low
2′-deoxyribose	4,5,6,7-tetrabromo-	high	moderate or high	moderate or high
	4,5,6,7-tetrachloro-	high	high	moderate or high
	4,5,6,7-tetraiodo-	high	moderate or high	moderate or high
	5,6-dibromo-	high	moderate or high	low
	5,6-dichloro-	high	moderate or high	low
	5,6-diiodo-	high	moderate or high	low
	5,6-dibromo,4,7-dichloro-	high	moderate or high	moderate or high
	5,6-dibromo,4,7-diiodo-	high	moderate or high	moderate or high
	5,6-diiodo,4,7-dibromo-	high	moderate or high	moderate or high
	5,6-diiodo,4,7-dichloro-	high	moderate or high	moderate or high
2′-deoxy-2′,2′-difluoro-ribose	4,5,6,7-tetrabromo-	high	moderate or high	moderate or high
	4,5,6,7-tetrachloro-	high	moderate or high	moderate or high
	4,5,6,7-tetraiodo-	high	moderate or high	moderate or high
	5,6-dibromo-	high	moderate or high	moderate or high
	5,6-dichloro-	high	moderate or high	moderate or high
	5,6-diiodo-	high	moderate or high	moderate or high
	5,6-dibromo,4,7-dichloro-	high	moderate or high	moderate or high
	5,6-dibromo,4,7-diiodo-	high	moderate or high	moderate or high
	5,6-diiodo,4,7-dibromo-	high	moderate or high	moderate or high
	5,6-diiodo,4,7-dichloro-	high	moderate or high	moderate or high

Table S4. Comparison of the binding modes of the individual ligands with respect to reference ligand the reference ligand.

glycone	Ligand	the entire complex			hydrophobic residues (Val66 and Lys174)		
		Euclidian	Manhattan	Additive	Euclidian	Manhattan	Additive
-	4,5,6,7-tetrabromo-*	-	-	-	-	-	-
	4,5,6,7-tetrachloro-	1.72	3.64	1.80	0.72	0.89	0.81
	4,5,6,7-tetraiodo-	2.49	4.68	-1.63	1.16	1.51	-1.51
	5,6-dibromo-	83.56	24.54	5.75	1.01	1.42	0.09
	5,6-dichloro-	92.74	26.15	7.13	1.48	1.70	0.64
	5,6-diiodo-	76.18	23.61	6.17	2.18	2.09	-.33
	5,6-dibromo,4,7-dichloro-	0.77	2.45	-0.15	0.45	0.95	-0.03
	5,6-dibromo,4,7-diiodo-	3.99	5.26	-1.64	0.63	1.06	-1.06
	5,6-diiodo,4,7-dibromo-	2.29	4.28	-1.66	1.17	1.52	-1.52
	5,6-diiodo,4,7-dichloro-	2.80	5.06	-2.16	1.02	1.43	-1.43
	native ligand	7.57	7.74	-2.786	-	-	-
ribose	4,5,6,7-tetrabromo-*	-	-	-	-	-	-
	4,5,6,7-tetrachloro-	4.76	6.29	1.66	1.00	1.42	1.10
	4,5,6,7-tetraiodo-	11.20	9.18	-4.23	0.10	0.53	0.16
	5,6-dibromo-	19.25	13.07	-12.47	2.30	2.53	-2.53
	5,6-dichloro-	18.37	12.36	-11.04	2.03	2.27	-2.27
	5,6-diiodo-	19.78	13.27	-12.47	3.89	3.29	-3.29
	5,6-dibromo,4,7-dichloro-	4.89	6.48	0.48	0.64	1.14	0.97
	5,6-dibromo,4,7-diiodo-	9.92	8.98	-4.53	0.55	1.02	0.65
	5,6-diiodo,4,7-dibromo-	7.43	7.56	-1.56	0.25	0.78	0.60
	5,6-diiodo,4,7-dichloro-	6.37	7.29	-0.60	0.19	0.63	0.44
	native ligand	23.32	10.85	1.74	-	-	-
2′-deoxyribose	4,5,6,7-tetrabromo-*	-	-	-	-	-	-
	4,5,6,7-tetrachloro-	2.13	3.74	2.11	0.02	0.19	-0.01
	4,5,6,7-tetraiodo-	2.95	4.71	-2.62	0.15	0.54	-0.14
	5,6-dibromo-	16.37	12.42	-9.37	0.99	1.39	-1.39
	5,6-dichloro-	17.60	12.93	-8.78	0.78	1.13	-1.13
	5,6-diiodo-	15.41	11.80	-9.77	1.11	1.46	-1.46
	5,6-dibromo,4,7-dichloro-	3.29	5.62	1.57	0.40	0.80	-0.41
	5,6-dibromo,4,7-diiodo-	6.63	6.87	-3.69	0.38	0.63	-0.63
	5,6-diiodo,4,7-dibromo-	6.69	7.21	-0.63	0.04	0.25	0.15
	5,6-diiodo,4,7-dichloro-	4.82	7.02	1.73	0.38	0.78	-0.41
	native ligand	29.37	13.93	3.29	-	-	-
2′-deoxy-2′,2′-difluoro-ribose	4,5,6,7-tetrabromo-*	-	-	-	-	-	-
	4,5,6,7-tetrachloro-	2.59	4.54	1.50	0.07	0.35	0.35
	4,5,6,7-tetraiodo-	6.70	7.41	0.50	2.53	1.84	1.84
	5,6-dibromo-	16.66	12.47	-11.60	2.93	2.37	-2.37
	5,6-dichloro-	13.58	11.26	-9.32	1.35	1.64	-1.64
	5,6-diiodo-	12.35	10.31	-9.88	0.87	1.31	-1.31
	5,6-dibromo,4,7-dichloro-	3.03	4.73	0.73	0.08	0.37	0.37
	5,6-dibromo,4,7-diiodo-	4.89	6.25	-3.73	0.02	0.18	0.18
	5,6-diiodo,4,7-dibromo-	0.50	1.69	-0.37	0.03	0.21	0.13
	5,6-diiodo,4,7-dichloro-	2.85	4.60	0.02	0.08	0.30	0.25

* reference ligand

Table S5. Comparison of the binding modes of the individual ligands with respect to the native ligand (atomic terms).

glycone	ligand	the entire ligand			halogen atoms (at 4,5,6,7 positions)		
		Euclidian	Manhattan	Additive	Euclidian	Manhattan	Additive
-	4,5,6,7-tetrabromo-*	-	-	-	-	-	-
	4,5,6,7-tetrachloro-	1.67	3.20	-1.20	1.43	2.10	-1.94
	4,5,6,7-tetraiodo-	1.73	3.60	-0.90	1.16	1.63	0.87
	5,6-dibromo-	97.68	28.40	16.22	15.77	5.54	-0.93
	5,6-dichloro-	100.06	28.07	16.56	13.57	4.91	-1.75
	5,6-diiodo-	108.29	30.01	-0.84	15.37	5.42	-1.19
	5,6-dibromo,4,7-dichloro-	0.99	2.64	-0.94	0.87	1.77	-1.24
	5,6-dibromo,4,7-diiodo-	1.18	2.87	-0.36	0.80	1.41	0.23
	5,6-diiodo,4,7-dibromo-	1.34	2.67	-0.65	1.04	1.44	0.42
	5,6-diiodo,4,7-dichloro-	1.96	4.14	-1.87	1.40	2.14	0.12
ribose	4,5,6,7-tetrabromo-*	-	-	-	-	-	-
	4,5,6,7-tetrachloro-	10.46	10.33	4.71	2.46	2.50	-1.94
	4,5,6,7-tetraiodo-	11.95	10.52	3.48	3.74	2.89	-2.85
	5,6-dibromo-	8.31	8.31	3.28	0.55	0.83	-0.83
	5,6-dichloro-	7.67	7.69	3.91	0.19	0.57	-0.57
	5,6-diiodo-	9.39	8.77	2.80	1.59	1.34	-1.34
	5,6-dibromo,4,7-dichloro-	10.16	10.05	5.87	1.82	2.21	-0.57
	5,6-dibromo,4,7-diiodo-	11.45	11.17	4.69	2.68	2.79	-2.79
	5,6-diiodo,4,7-dibromo-	11.37	9.97	5.30	3.10	2.32	-1.21
	5,6-diiodo,4,7-dichloro-	11.66	10.64	5.55	3.65	2.95	-0.53
2′-deoxyribose	4,5,6,7-tetrabromo-*	-	-	-	-	-	-
	4,5,6,7-tetrachloro-	1.29	2.76	-1.63	1.14	1.60	-1.60
	4,5,6,7-tetraiodo-	0.81	2.93	-1.12	0.66	1.57	-0.92
	5,6-dibromo-	3.52	6.38	1.26	0.18	0.59	-0.10
	5,6-dichloro-	4.51	7.33	1.31	1.12	1.46	-0.34
	5,6-diiodo-	3.77	6.49	0.62	0.37	0.65	-0.65
	5,6-dibromo,4,7-dichloro-	2.57	5.71	1.20	0.54	1.07	-1.05
	5,6-dibromo,4,7-diiodo-	5.23	7.26	-0.56	3.53	3.00	-3.00
	5,6-diiodo,4,7-dibromo-	3.72	6.41	0.52	2.20	2.33	-1.68
	5,6-diiodo,4,7-dichloro-	3.15	5.90	1.36	1.18	1.56	-0.52
2′-deoxy-2′,2′-difluoro-ribose	4,5,6,7-tetrabromo-*	-	-	-	-	-	-
	4,5,6,7-tetrachloro-	2.65	5.10	0.05	1.79	2.05	-2.01
	4,5,6,7-tetraiodo-	2.63	5.30	1.53	1.09	1.80	-1.25
	5,6-dibromo-	0.59	2.79	-2.35	0.02	0.20	-0.04
	5,6-dichloro-	1.04	4.12	-3.02	0.20	0.58	-0.58
	5,6-diiodo-	1.59	4.98	-3.35	0.42	0.88	-0.24
	5,6-dibromo,4,7-dichloro-	1.80	4.53	1.49	0.85	1.30	-0.47
	5,6-dibromo,4,7-diiodo-	2.52	5.24	0.73	1.54	2.25	-1.93
	5,6-diiodo,4,7-dibromo-	1.00	3.44	1.20	0.50	1.18	-0.05
	5,6-diiodo,4,7-dichloro-	3.16	5.35	1.18	2.37	2.47	-0.27

* reference ligand

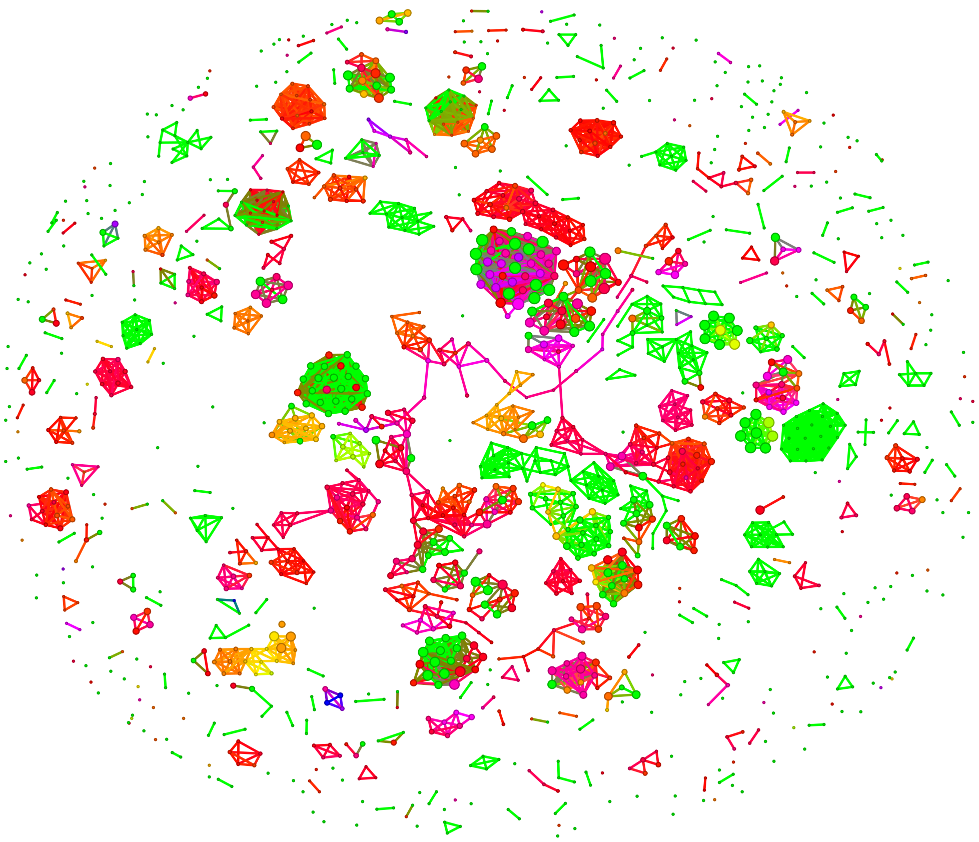

Figure S1. A SBAI diagram, with Skeleton Spheres as similarity measure, for the set of studied ligands (validation task).
